# Synthesis, Growth Mechanism, and Photocatalytic Properties of Metallic-Bi/Bi_13_S_18_Br_2_ Nano-Bell Heterostructures

**DOI:** 10.1021/acsmaterialslett.5c00043

**Published:** 2025-04-01

**Authors:** Anna Cabona, Stefano Toso, Andrea Griesi, Martina Rizzo, Michele Ferri, Pascal Rusch, Giorgio Divitini, Julia Pérez-Prieto, Raquel E. Galian, Ilka Kriegel, Liberato Manna

**Affiliations:** † Nanochemistry Department, Italian Institute of Technology, Via Morego 30, 16163 Genova, Italy; ‡ Department of Applied Science and Technology, Politecnico di Torino, Corso Duca degli Abruzzi 34, 10129 Turin, Italy; § Lund University, Division of Chemical Physics, Naturvetarvägen 14, 221 00 Lund, Sweden; ∥ Electron Spectroscopy and Nanoscopy, Italian Institute of Technology, Via Morego 30, 16163 Genoa, Italy; ⊥ Institute of Molecular Science, University of Valencia, c/Catedrático José Beltrán Martínez 2, 46980 Paterna, Valencia, Spain

## Abstract

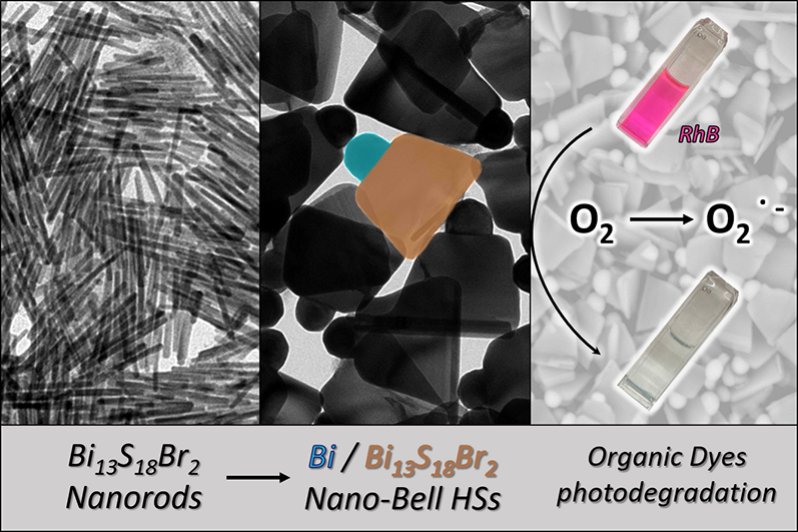

We report the synthesis of bell-shaped Bi/Bi_13_S_18_Br_2_ metal/semiconductor heterostructures as a photocatalyst based on nontoxic and Earth-abundant elements. Their unique morphology arises from a multistep growth process, involving (1) the nucleation of Bi_13_S_18_Br_2_ nanorods, (2) the reduction of a metallic-Bi domain on their surface induced by *N,N*-didodecylmethylamine, and (3) the heterostructure accretion by a localized reaction at the Bi/Bi_13_S_18_Br_2_ interface promoted by Ostwald ripening. These heterostructures display remarkable stability in polar solvents, remaining almost unaffected by prolonged exposure to isopropanol and water, and exhibit high photocatalytic efficiency for the degradation of organic dyes (i.e., Rhodamine B and Methylene Blue) under visible-light irradiation, with good recyclability. Additionally, preliminary tests demonstrate CO_2_ reduction capabilities, which make these heterostructures promising for both the photocatalytic degradation of pollutants and photoelectrochemical CO_2_ conversion. The straightforward synthesis process and the use of nontoxic and Earth-abundant elements offer significant potential for sustainable energy conversion technologies.

Colloidal nanoheterostructures enable combining the properties of different materials into a single functional nanocomposite and may lead to the emergence of new properties stemming from the interaction of the two materials.^1−4^ Among the various heterostructures reported to date, those composed of a metal and a semiconductor stand out as promising candidates for light harvesting applications, as the semiconductor domain can convert light into electron–hole pairs, and the metal domain makes them easy to access. Relevant examples include Au-CdSe,^5^ Pt-CdS,^6^ and M-CsPbBr_3_ (M = Pd,Pt) heterostructures,^7^ which exhibit promising performance as light-harvesters,^8−10^ photodetectors,^11^ and photocatalysts.^12−14^

A class of semiconductors recently considered in the preparation of colloidal heterostructures are metal chalcohalides,^15−17^ which can be synthesized using methods and chemicals similar to those employed for metal halides (e.g., CsPbBr_3_) but do not suffer from the intrinsic lability of metal halides.^18,19^ Among them, bismuth-based chalcohalides^20−22^ are noteworthy due to their low cost and low toxicity, promising photovoltaic^23^ and thermoelectric^24−26^ properties, and well-established solid-state chemistry.^27,28^ Colloidal synthesis routes have been proposed for two bismuth chalcohalide phases: the orthorhombic BiSX (X = Cl, Br, I)^19^ and the hexagonal Bi_13_S_18_X_2_ (X = Br, I).^30^ In parallel, reports on CsPbBr_3_/PbBi_2_S_4_ heterostructures have shown how semiconductors containing bismuth and sulfur can be integrated in colloidal heterostructures.^31,32^

In this work, we demonstrate the growth of metallic-Bi/Bi_13_S_18_Br_2_-chalcohalide heterostructures exhibiting a bell-shape (Figure 1a). This was made possible by introducing substantial modifications in the synthesis procedure for bismuth chalcohalide nanocrystals, originally developed by Quarta et al. in collaboration with some of us.^19^ In that work, nanocrystals were grown by reacting a Bi-oleate solution with different amounts of sulfur and halide precursors (e.g., trimethylsilyl sulfide and bromide). The type of halide and precursor ratio could be adjusted to obtain either the orthorhombic BiSX or the hexagonal phase Bi_13_S_18_X_2_, both of which formed nanorods due to their anisotropic crystal structures, characterized by tubular Bi–S networks. Notably, the original protocol avoided the use of amines as surfactants to prevent the reduction of Bi^3+^ to metallic Bi (Figures S1 and S2).

**Figure 1:**
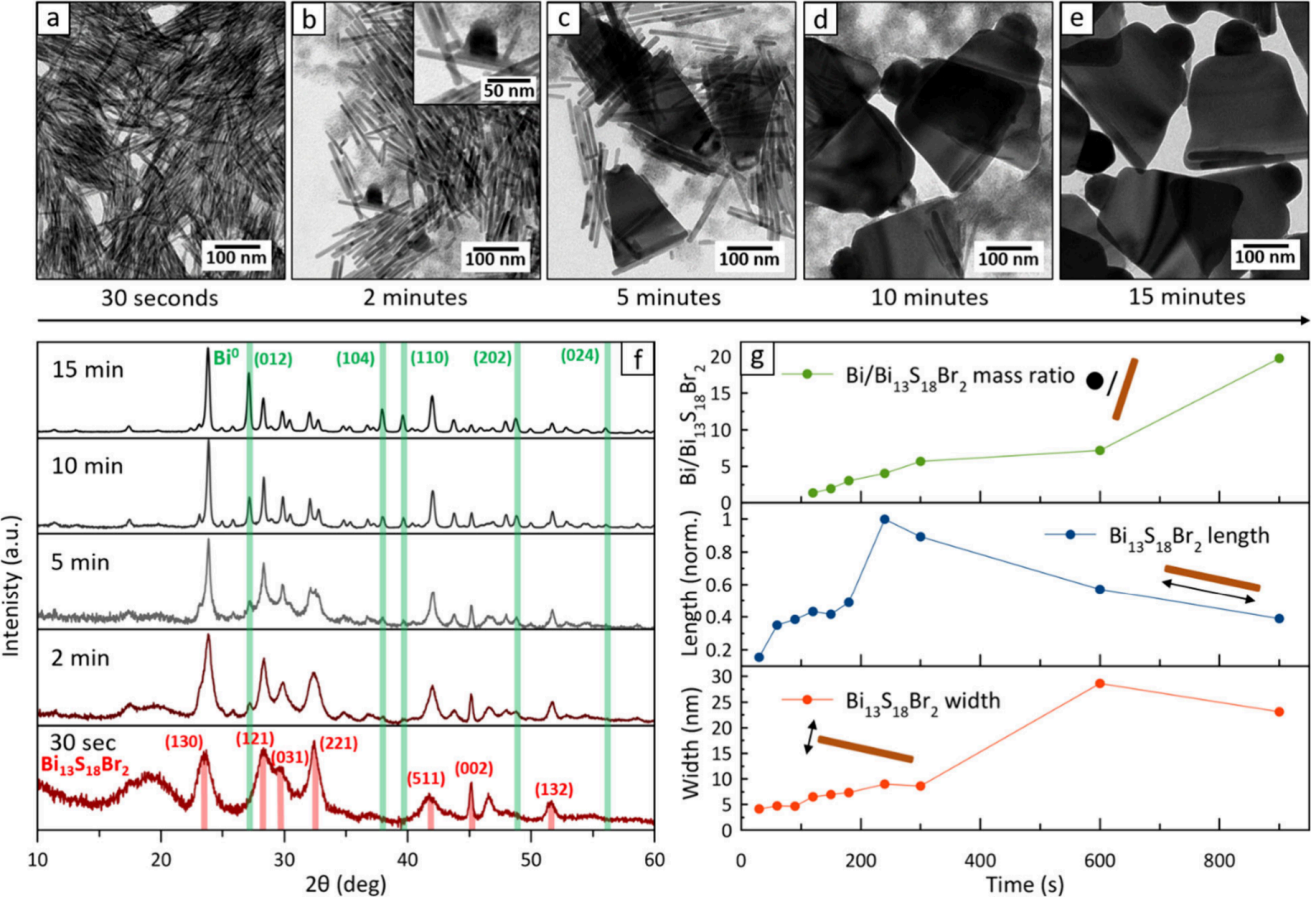
Synthesis of Bi/Bi_13_S_18_Br_2_ nanobells. (a–e) TEM images of aliquots collected at 0.5, 2, 5, 10, and 15 min from the start of the reaction. The inset in panel (b) highlights an early stage Bi/Bi_13_S_18_Br_2_ heterostructure, where the chalcohalide domain is still rod-shaped. (f) XRD patterns of the aliquots, showing a progressive enlargement of the chalcohalide domains (peaks becoming sharper) and the growth of metallic-Bi domains (additional peaks appearing, marked in green). (g) Compositional and morphological descriptors extracted from the Rietveld fit of XRD profiles. From top to bottom: Bi/Bi_13_S_18_Br_2_ mass ratio (metallic Bi is not detectable in the first three patterns, top); average Bi_13_S_18_Br_2_ crystallites length along the *c* lattice direction (rod length, middle); average Bi_13_S_18_Br_2_ crystallites thickness perpendicular to the *c* lattice direction (bottom).

In the present work, we speculated that controlling this reduction process could enable the growth of functional metal–semiconductor heterostructures. Therefore, we modified the synthetic protocol for the orthorhombic BiSBr nanocrystals by injecting oleylamine into the reaction mixture just before adding the sulfur and bromine precursors. Contrary to expectations, these initial tests yielded Bi_13_S_18_Br_2_ nanorods instead of BiSBr. Among them, we observed the occasional formation of peculiar heterostructures consisting of a triangular sheet of Bi_13_S_18_Br_2_ with a spherical, metallic bismuth cap attached to it (Figures S3 and S4), and which in projection appeared as bell-shaped.

The nucleation of Bi_13_S_18_Br_2_ instead of BiSBr is consistent with a more reducing environment, as this compound contains subvalent [Bi–Bi]^4+^ dimers.^30,35,36^ However, the introduction of a primary amine did not lead to the formation of metallic bismuth, as initially expected. To address this, we replaced it with a tertiary amine (*N,N*-didodecylmethylamine), driven by the hypothesis that the inductive effect of the alkyl chains would make it a stronger nucleophile and a better reducing agent, capable of inducing the heterogeneous nucleation of bismuth on the surface of Bi_13_S_18_Br_2_ nanorods.

Figure 1a–e illustrates the morphological evolution of the resulting particles tracked by extracting aliquots at different time steps. In the early stages of the reaction (30 s to 1.5 min, see Figure S5a–h) only Bi_13_S_18_Br_2_ rods were visible under the transmission electron microscope (TEM). These rods were found to nucleate just after the injection of precursors, and their length steadily increased in the early phases of the reaction (Figure S6). However, after 2 min, metallic-Bi domains began to form at the center of some nanorods, appearing as large and dark hemispheres (Figure 1b). As the reaction proceeded, the number of free nanorods progressively decreased while the metal-rod heterostructures grew wider, ultimately acquiring their characteristic bell shape. By 15 min, no free nanorods had remained in the sample (Figure 1d, e, see also Figures S6–S8).

Rietveld fits of X-ray diffraction (XRD)^37^ patterns collected from the same aliquots (Figure 1f, see also Figure S9) supported the conclusions from electron microscopy. The presence of metallic bismuth became detectable after 2 min, and its estimated relative fraction increased linearly during the reaction (Figure 1g, top). Similarly, the average lateral size of Bi_13_S_18_Br_2_, extracted by matching the width of peaks with an anisotropic spherical harmonics model of the crystallite shape,^19,38,40^ increased from the initial estimate of 4 nm up to ∼25 nm. The rod length initially increased as well (up to ∼4 min), indicating that the accretion of the Bi_13_S_18_Br_2_ nanorods was still ongoing. However, starting from ∼5 min the length of such isolated nanorods dropped quickly as they started to be consumed in favor of the nanobells, likely by an Ostwald ripening process. Note that the rod length estimates from XRD are not quantitative, as it is challenging to model strongly anisotropic and flexible particles along their longest direction.^19^ However, a relative length comparison is valid, and the same morphological evolution was confirmed by TEM imaging, albeit with lower statistics (Figure S10).

Scanning-transmission electron microscopy images (STEM, Figure 2a) and energy-dispersive X-ray spectroscopy (EDX, Figure 2b) maps of the heterostructures evidenced that Bi, S and Br atoms are uniformly distributed in the chalcohalides domain (Figure S11) and revealed the presence of an ∼1.3 nm Br-rich layer on the surface of the metallic-Bi hemisphere. This layer grows thicker (∼7–8 nm) at the neck region connecting the two domains and is likely polycrystalline (Figure S12). These observations are congruent with the elemental composition observed in each domain by EDX analysis (Figure S13). Further inspection of the interface by high-resolution (HR)-STEM and 4D-STEM suggested an epitaxial relation between the two domains, as indicated by the matching periodicity of the lattice fringes at the interface and the presence of nearly aligned atomic planes (Figure 2c). A plausible epitaxial relation is (001)//(1–10) – Bi/Bi_13_S_18_Br_2_, which was identified by feeding the probable lattice orientations inferred by 4D-STEM into the lattice matching module of the Ogre library for the prediction of epitaxial interfaces,^42^ developed by the Marom group in collaboration with some of us (Figure 2d, see Figure S14 for further discussion).

**Figure 2:**
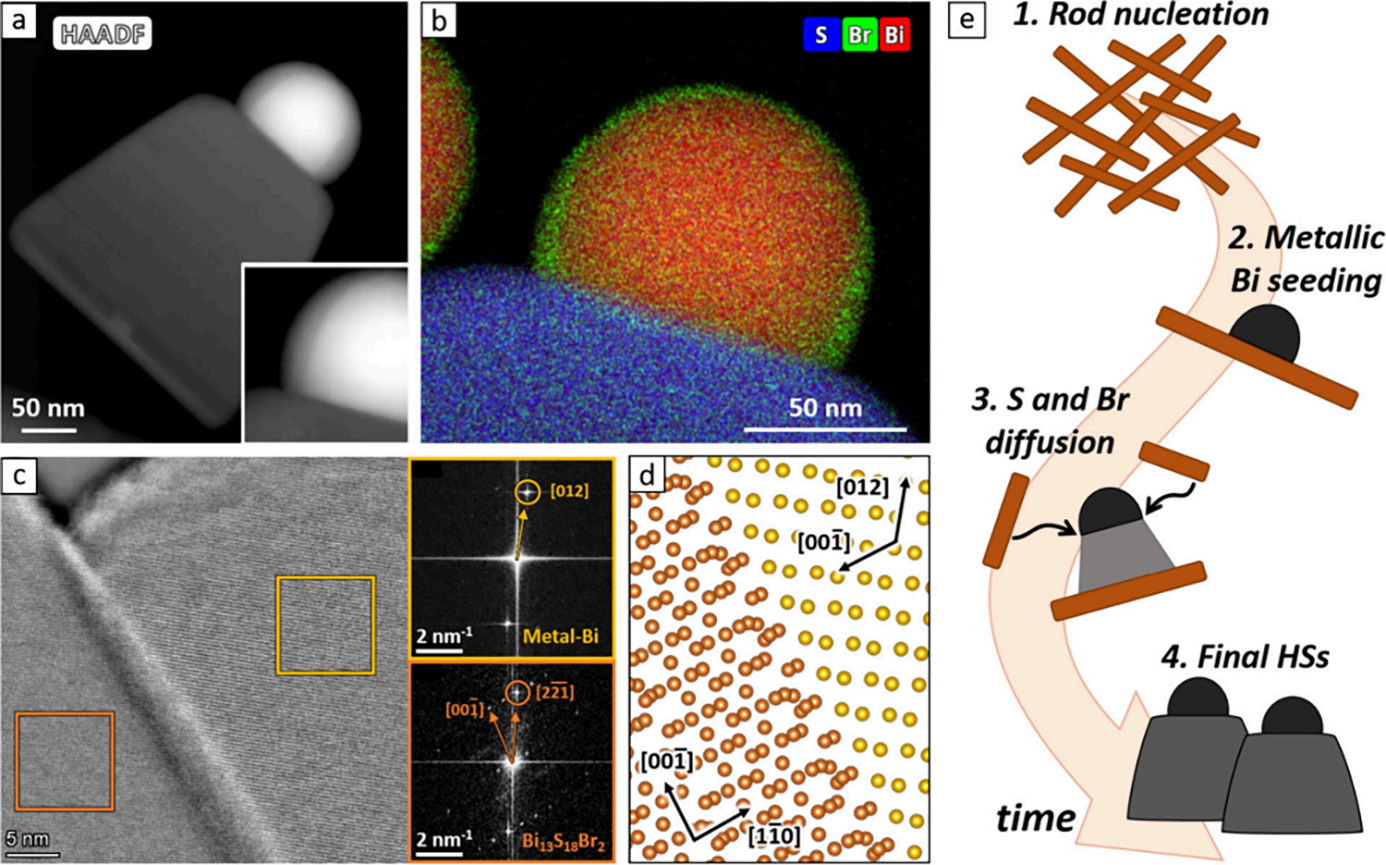
Composition and structure of Bi/Bi_13_S_18_Br_2_ nanobells. (a) HAADF image of one heterostructure. b) STEM-EDX compositional map of the Bi/chalcohalide contact region, showing the presence of a Br-rich shell surrounding the metal hemisphere. (c) HAADF image of the Bi/Bi_13_S_18_Br_2_ interface region, with lattice fringes enhanced by a high-pass filter. Insets: Fourier transforms of the yellow and orange areas, respectively. The circled spots identify the most prominent lattice planes visible along this orientation. (d) Proposed Bi/Bi_13_S_18_Br_2_ epitaxial model of the interface, with the two domains oriented in accordance with panel (c). Only bismuth atoms are shown for clarity, and the relative positions of the two domains are estimated (i.e., no model optimization performed). (e) Proposed formation mechanism for the Bi/Bi_13_S_18_Br_2_ nanobells.

When combined, these observations allow outlining a possible growth mechanism for the Bi/Bi_13_S_18_Br_2_ nanobells (Figure 2e). Initially, the injection of sulfur and bromine precursors triggers the nucleation of Bi_13_S_18_Br_2_ nanorods driven by the reactivity of the precursors and the ionic nature of the compound. Shortly thereafter, the amine starts to reduce Bi^3+^ to metallic bismuth, a process facilitated by the nanorods serving as heterogeneous nucleation templates. This reduction proceeds gradually due to the tertiary amine’s weak reducing ability and its consumption during the reaction.

Once the Bi/Bi_13_S_18_Br_2_ interface forms, it can act as a sink for excess anions. Bromine is already found at the surface of the metal hemisphere, and sulfur is adsorbed to produce more Bi_13_S_18_Br_2_ at the interface. This enlarges the chalcohalide sheet and gradually displaces the original nanorod (now forming the bottom edge of the bell) away from the bismuth hemisphere. Similar growth mechanisms are well documented in nanofabrication via molecular beam epitaxy, such as the use of Au nanoparticles to drive the grow of GaAs nanopillars,^43−45^ but remain rare in colloidal heterostructures.^46^ The process continues until all of the precursors in solution are depleted (∼5 min). At this point, Ostwald ripening drives further transfer of material from the nanorods to the heterostructures. As a result, the remaining metal-free nanorods gradually shrink and eventually disappear, leaving behind only Bi/Bi_13_S_18_Br_2_ nanobells at the end of the reaction.

To further validate this mechanism, we combined two reaction batches, one where the bells were allowed to fully develop and another that was quenched at the nanorods stage. The mixture was heated back to 180 °C to resume the reaction, which proceeded for an additional 15 min. As expected, the nanorods from the second batch did not develop into new, small heterostructures; instead, they were consumed to further enlarge the existing ones (see Figure S15).

We note that the epitaxial relation proposed in Figure 2d should cause an expansion of the chalcohalide in the plane of the interface (Figure S14). A 4D-STEM analysis, performed by capturing local electron diffraction patterns while scanning across one heterostructure, confirmed the presence of a strain field propagating from the Bi/Bi_13_S_18_Br_2_ interface. Figure 3b–e shows the linear strain components (ϵ_*xx*_ and ϵ_*yy*_), along with the shear strain and local lattice rotation (ϵ_*xy*_ and θ), reconstructed with py4DSTEM.^47^ The analysis confirms an ϵ_*yy*_ expansion of the Bi_13_S_18_Br_2_ lattice in the interface plane and a corresponding orthogonal ϵ_*xx*_ compression to keep the unit cell volume unchanged, in agreement with our predictions. Interestingly, while performing a HAADF tilt series, we also found that the darker domain often visible in TEM at the bottom of the bells is not a local thickening, but rather a curl that forms as the chalcohalide sheet rolls up at its bottom edge (Figures 3f). We speculate that this behavior might result from the need to accommodate some of the interface strain and is likely facilitated by the hexagonal crystal structure of Bi_13_S_18_Br_2_, which allows the domain to expose the same surface at the interface after a 60° rotation.

**Figure 3:**
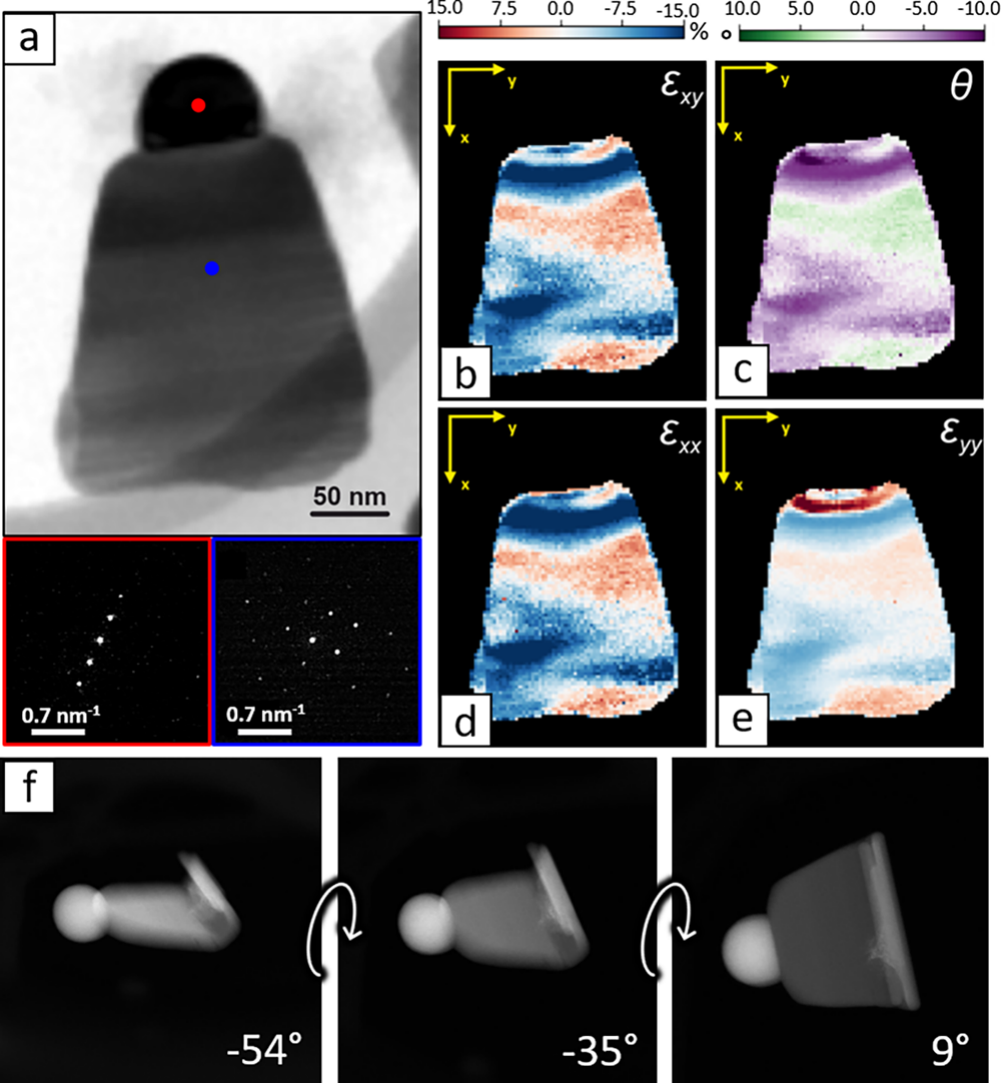
4D-STEM and tomography analysis. (a) Virtual bright field image reconstructed from the diffraction patterns of Bi and Bi_13_S_18_Br_2_ collected at each pixel. (b,c) Map of the shear strain and rotation of the lattice. (d,e) Bi_13_S_18_Br_2_ strain field components along the x- and *y*-directions. The *x* and *y* axes in panels (b–e) indicate the image reference system and are not related to crystallographic directions. (f) Frames from an HAADF tilt series, showing the curling of the lower section of a bell.

Owing to the 1.65 eV band gap^48^ of Bi_13_S_18_Br_2_, its high absorption coefficient,^30^ and its intimate connection with the metallic-Bi domain, these heterostructures are interesting candidates for converting visible light into highly accessible photocarriers. This motivated us to screen the photocatalytic activity of Bi/Bi_13_S_18_Br_2_ nanobells. As a model reaction, we selected the photodegradation of Rhodamine B (RhB), for which other Bi-based semiconductors are known to be active.^50,51^ The reaction was conducted in isopropanol under 420 nm irradiation (see the Methods), resulting in the complete degradation of RhB after 1 h. Control tests performed in the absence of heterostructures confirmed that the intrinsic photodegradation of the dye was negligible under the conditions adopted (4.5%, Figure S16). Likewise, tests performed under dark conditions, i.e., letting the RhB surface adsorption equilibrium establish, and then removing the particles resulted in a negligible decrease of the absorption signal (<1% RhB adsorption on the HS, Figure S17), confirming that the observed dye degradation was indeed the result of a photocatalytic process mediated by the Bi/Bi_13_S_18_Br_2_ heterostructures.

The photocatalytic mechanism was further investigated by repeating the reaction in the presence of benzoquinone, which acts as a scavenger for the superoxide anion (O_2_^.–^).^52^ Under these conditions, the RhB photodegradation was reduced to only 5.4% (Figure S18), highlighting a key role of O_2_^.–^ in the process. Indeed, a second test conducted in oxygen-free conditions to prevent the superoxide anion formation (N_2_ atmosphere, see Figure S19) resulted in almost no photodegradation of RhB. Note that we excluded the hydroxyl radical (·OH) as a possible reactive species, because isopropanol is an efficient ·OH scavenger.^53,54^Figure S20 summarizes the result of control experiments performed in the presence of N_2_ and benzoquinone, in the absence of photocatalyst, and in the dark.

Based on this evidence, we propose the reaction mechanism illustrated in Figure 4a (inset). First, in line with the Bi/Bi_13_S_18_Br_2_ band alignment, the photogenerated electron moves to the metallic-Bi domain, which acts as a cocatalyst. There, the electron is unlikely to recombine with the hole, which remains localized on the chalcohalide domain and becomes accessible to reduce one molecule of O_2_ to the O_2_^.–^ superoxide anion, which then oxidizes the substrate.^55,56^ The fate of the hole is uncertain: it may be consumed by reducing the 2-propanol or may further activate the superoxide by transforming it into singlet-oxygen (^1^O_2_), which is itself a highly reactive oxidizer. The effectiveness of benzoquinone as an O_2_^.-^ scavenger reveals that this is the main reactive oxygen species (ROS) responsible for photodegradation of RhB. The photodegradation mechanism operated by this reactive oxygen species (ROS) could involve N-de-ethylation, chromophore cleavage, and eventually mineralization steps. Based on the hipsochromic shift observed in the absorption spectra during the irradiation time (Δλ = 16 nm at 45 min), we can hypothesize the N-de-ethylation of the dye with the formation of a series of N-de-ethylated intermediates as a possible mechanism. This could be followed by the cleavage of the chromophore structure, suggesting a stepwise breakdown of the molecular structure of the dye, in agreement with previous reports.^57^ On the other hand, the use of IPA as solvent ruled out the participation of OH· radicals and holes (h^+^) in the mechanism, as it is a well-known scavenger of both species.^58^

**Figure 4:**
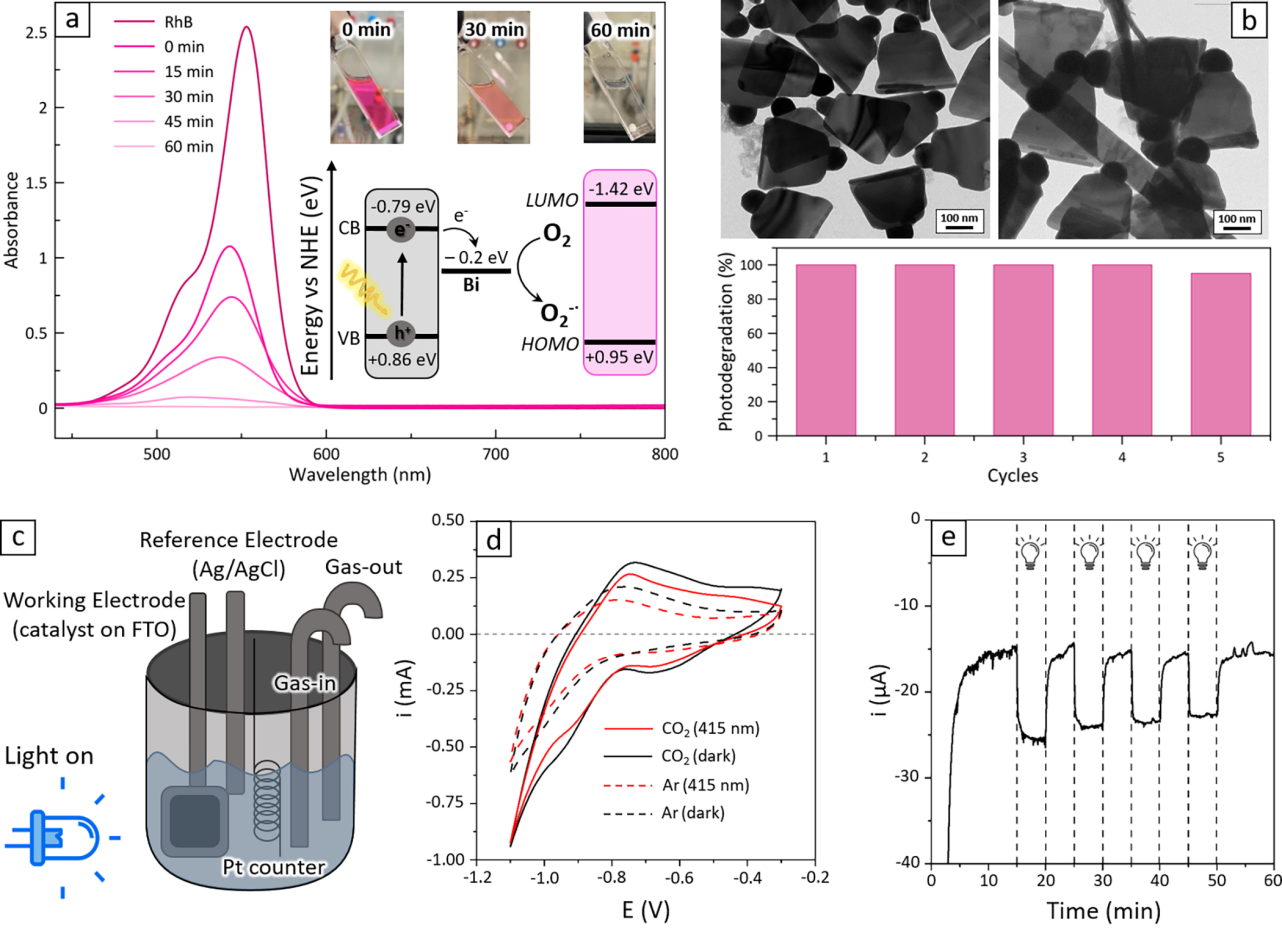
Photo- and photoelectrocatalytic activity of Bi/Bi_13_S_18_Br_2_ heterostructures. (a) Absorption spectra and pictures (insets) of the RhB solution taken at various stages of the photodegradation reaction (top) and scheme of the proposed photodegradation mechanism (down). (b) TEM images of Bi HSs before (left) and after (right) 5 cycles of RhB photodegradation (top); photodegradation activity for the five cycles (down). (c) Scheme of the cell used for the photoelectrochemical testing of heterostructure-based electrodes. (d) Cyclic voltammetry traces collected on the heterostructures (*v* = 100 mV s^–1^). The dashed curves were collected under Ar bubbling (blank tests; only hydrogen evolution reaction is possible), while the full traces are related to CO_2_ reduction reaction tests. Black: dark conditions. Red: 415 nm LED illumination. (e) Chronoamperometric scan, performed at −1 V vs the reference electrode under intermittent illumination.

Further tests performed with methylene blue showed similar results to RhB, with 93% degradation after 2 h (Figures S21–23), while no degradation was observed for methyl orange (Figure S24). This difference is attributed to a poor affinity of the anionic methyl-orange for the nanobell surface compared to cationic dyes like RhB and methylene blue, which is consistent with the measured negative Zeta potential value (−58.3 mV, see Table S1).

Notably, the heterostructures maintained almost full photocatalytic activity (ca. 95% degradation of the selected dyes) after 5 cycles (Figure 4b), with virtually no catalyst degradation and only minor signs of aggregation despite the prolonged exposure to a polar environment. Although the colloidal stability of the nanocrystals is limited due to their large size, a postreaction TEM analysis showed no noticeable alteration of the material, further attesting its robustness (Figure 4b). This stability underscores the potential of these heterostructures for practical applications, where durability and consistent performance are required.

Motivated by this remarkable durability, we attempted a preliminary test for the activity of our Bi/Bi_13_S_18_Br_2_ heterostructures in the CO_2_ reduction reaction (CO_2_RR). Indeed, metallic-Bi is known to be a good catalyst for CO_2_RR, and the heterostructures’ resilience to a polar solvent like IPA suggested that they could withstand water.

The CO_2_RR tests were conducted using our previously reported electrochemical CO_2_RR setup.^59^ Briefly, photoelectrodes were crafted by spin coating the heterostructures on fluorine-doped tin-oxide (FTO) glass and were installed in a three-electrode, single-compartment quartz cell (see Figure 4c and methods for setup, electrochemical testing, and product quantification). Preliminary Open Circuit Voltage (OCP, Figure S25) measurements performed under intermittent 415 nm illumination revealed a photoresponse in the form of a reversible shift toward more cathodic values upon illumination. Conversely, Cyclic Voltammetry (CV)^60^ displayed limited sensitivity to light in terms of current/voltage relationship (black versus red traces in Figure 4d). For reference, a comparable signal was measured from the bare FTO control electrode (Figure S26).

Nevertheless, clear differences in the CV traces emerged when CO_2_ was introduced into the setup, indicating catalytic activity. As shown by the dashed traces in Figure 4d (collected under Ar flow), a smooth trace with a clear onset potential (*E*_Onset_) around −0.8 V versus RE can be observed and is attributed to the Hydrogen Evolution Reaction (HER). In contrast, when CO_2_ is present (full lines in Figure 4d), the voltammogram displays additional features (peaks/waves at ca. −0.6 and −1 V vs RE) that are consistent with the CO_2_ preadsorption and reduction onset. This hypothesis is supported by the increased current density recorded at large cathodic potentials, which indicates a CO_2_RR contribution to the HER current observed in Ar-fed tests.

In an attempt to identify parasitic HER and CO_2_RR product distribution and Faradaic efficiency, we also performed a 1 h long ChronoAmperometric (CA) scan at −1 V vs RE under CO_2_RR conditions. The intermittent illumination (5 min-long on/off cycles, Figure 4e) clearly enhanced the reaction rate (i.e., current density), further proving the CO_2_RR photoelectroactivity of heterostructures, as the same photoresponse was not observed during control tests (Figure S27). However, the low current densities translated into limited production rates, making it challenging to detect and quantify both the HER and the CO_2_RR products (see Figure S28 and related discussion).

In conclusion, we have reported a synthetic protocol to obtain a Bi/Bi_13_S_18_Br_2_ metal/semiconductor heterostructure through the controlled introduction of a reducing agent. These heterostructures adopt a unique bell-like morphology, resulting from a complex, multistage nucleation process: the formation of initial Bi_13_S_18_Br_2_ nanorods, followed by the growth of metallic-Bi on their surface, and finally by an interfacial accretion process driven by Ostwald ripening. These heterostructures demonstrate remarkable stability under operando conditions, even when exposed to polar solvents like isopropanol and water. They also show high photocatalytic activity, good recyclability under visible-light irradiation for cationic dye photodegradation, and preliminary CO_2_ reduction capabilities. Their ease of synthesis, use of nontoxic and earth-abundant elements, and promising performances highlight their potential for future applications in and beyond photocatalysis.

## Experimental Section

### Chemicals

All chemicals were of the highest purity available, unless otherwise noted, and were used as received. Bismuth(III) acetate (Bi(Ac)_3_; 99.99%), oleic acid (technical grade, 90%), 1-octadecene (technical grade, 90%), bis(trimethylsilyl)sulfide ((Me_3_Si)_2_S; synthesis grade), and trimethylsilyl bromide ((Me_3_Si)Br; 97%) were purchased from Sigma-Aldrich. *N,N*-Didodecylmethylamine (85%) was purchased from TCI. Hexane and toluene were purchased from Sigma-Aldrich. All solvents were purchased anhydrous and were used as received.

### Synthesis of Bi/Bi_13_S_18_Br_2_ Nanobells

The synthesis is a modification of the method already reported by Quarta et al.^19^ In short: all colloidal nanocrystals (NCs) were synthesized in three-neck flasks attached to a standard Schlenk line, ensuring oxygen- and moisture-free conditions. In a typical procedure to obtain Bi/Bi_13_S_18_Br_2_ NCs, 0.3 mmol (120 mg) of Bi(Ac)_3_ and 6.3 mmol (2 mL) of oleic acid were combined in 3 mL of 1-octadecene. The mixture was stirred vigorously and deoxygenated through several cycles of vacuum and nitrogen purging at around 80 °C. Subsequently, the mixture was heated to 110 °C to fully dissolve Bi(Ac)_3_, which caused the solution to turn colorless and optically clear, indicating the complete formation of the bismuth(III)-oleate complex(es). The solution was maintained under vacuum at 110 °C for 30 min to eliminate any acetic acid formed during the complexation process. Afterward, the solution was reheated under nitrogen flow, with the temperature stabilized at 180 °C. At this stage, 300 μL of didodecylmethylamine was injected, followed immediately by the coinjection of a half equivalent of (Me_3_Si)_2_S (0.15 mmol; 32 μL) and one equivalent of (Me_3_Si)Br (0.3 mmol; 39.5 μL) dissolved in 2 mL of octadecene. The reaction was allowed to proceed for 15 min before the heating source was removed. The colloidal dispersion was quickly cooled to room temperature by placing the reaction flask in an ice bath. After the reaction, the mixture was centrifuged at 6000 rpm for 5 min. The clear supernatant was discarded, and the resulting black precipitate was collected in 1 mL of toluene or hexane. No additional purification steps were carried out on the NC heterostructures.

### Electron Microscopy

Bright Field Transmission Electron Microscopy (BF-TEM) measurements of the NCs were performed using either a JEOL JEM-1011 with a W thermionic source at an acceleration voltage of 100 kV or a JEOL JEM-1400Plus TEM, with LaB_6_ thermionic source and maximum acceleration voltage 120 kV. The highly diluted NC solution was first put in ultrasound for 1 min and then drop-cast onto copper grids (200 mesh) with carbon film, and the solvent was then allowed to evaporate in a vapor-controlled environment. The longitudinal and lateral dimensions were assessed through statistical analysis of TEM images of several hundred NCs using the ImageJ software. Selected area electron diffraction measurements were performed on the same microscope and evaluated using the CrysTBox software package.

High-resolution scanning transmission electron microscopy (HRSTEM) images were acquired on a probe-corrected Thermo Fisher Spectra 300 STEM operated at 300 kV. Images were acquired on a high-angle annular dark-field (HAADF) detector with a current of ∼100 pA. Compositional maps were acquired and analyzed using Velox, with a probe current of ∼150 pA and rapid rastered scanning, on a Dual-X detector setup. 4DSTEM data were collected on a Gatan Continuum using STEM-X, with a current of ∼5 pA and a dwell time of 1 ms. The analysis of the 4DSTEM data sets was carried out using the open-source Python code py4DSTEM.^47^

### X-ray Powder Diffraction

Characterization by powder X-ray diffraction (XRD) was performed by employing a PANalytical Empyrean X-ray diffractometer using a 1.8 kV Cu Kα ceramic X-ray tube operating at 45 kV and 40 mA and detected by a PIXcel3D 2 × 2 area detector. Samples were prepared by drop-casting highly concentrated solutions on zero-diffraction silicon substrates. All diffraction patterns were acquired at room temperature under ambient conditions. Data analysis was performed using HighScore 4.9 software from PANalytical. The Rietveld fits of XRD patterns were performed with the FullProf suite,^40^ and were specifically oriented to the extraction of morphological information and mass ratios. To this end, additional effort was devoted to the appropriate modeling of the peak intensities and profiles. The instrumental broadening was quantified by measuring and fitting a LaB_6_ diffraction pattern (not shown) to construct the instrumental response function. Then, the pattern was fitted by refining for both phases (metallic Bismuth and Bi_13_S_18_Br_2_) the following parameters: scale factor, lattice parameters (*a, b, c*, whereas angles were fixed by symmetry), polynomial background, instrumental zero, and spherical harmonics size parameters (1 parameter for metallic bismuth = spherical crystallite, up to 3 parameters for Bi_13_S_18_Br_2_ = Y00, Y20, Y40). March-Dollase modeling for the preferred orientation of Bi_13_S_18_Br_2_ was included to account for the possible orientation of the large and flat nanobells upon deposition on the substrate, to correctly account for the observed intensity of reflections. Instead, the position of atoms was not refined to avoid the introduction of unjustified distortion to the crystal structure, which would likely mask the actual underlying microstructural contributions to the pattern. In those cases where the Bi domain was close to its detection limit, the domain size was fixed to the last value that could be determined reliably to avoid overfitting.

### Photocatalysis Tests

For the photodegradation of the organic dyes, a 1 mM solution of MB or RhB was prepared in 20 mL of IPA, in 20 mL of methanol in the case of MO. Then, 2 mg of Bi/Bi_13_S_18_Br_2_ NCs were mixed with 73 μL of the organic solution until a final volume of 2.5 mL of IPA in quartz cuvettes. Subsequently, the cuvettes were introduced in a photoreactor (λ_ex_ = 420 nm) until the complete photodegradation of the organic pollutants, measuring the UV–vis spectra for following the process at different photoreaction times. The solution containing the photocatalyst and the organic molecule was centrifuged (7000 rpm for 5 min), and the spectra were collected from the supernatant. UV–visible absorption spectra were recorded on a spectrophotometer UV/vis/NIR Lambda 1050, equipped with software PerkinElmer UV Winlab. Quartz cuvettes of 1 cm × 1 cm path length were used to acquire all the data. As a photoreactor was used, the PhotoreactorM2 from Penn PhD, with stirring at 400 rpm and the fan at 2800 rpm. For the study of the reaction mechanism, a 1 mM solution of benzoquinone (scavenger) was prepared in 1 mL of IPA. Then, 2 mg of Bi/Bi_13_S_18_Br_2_ NCs were mixed with 73 μL of RhB solution and 100 μL of BZQ solution until a final volume of 2.5 mL of IPA in quartz cuvette. Before the irradiation, the mixture was stirred for 15 min in the dark to establish the adsorption–desorption equilibrium of RhB on the photocatalyst surface. The zeta (ζ) potential was determined by employing a Zetasizer Ultra instrument (Malvern, UK).

### Photoelectrochemical Tests

#### Photoelectrode Preparation

1 × 1 cm electrodes were crafted starting from the toluene suspensions of Bi HSs and Bi rods obtained from the synthesis. They were obtained by spin coating (Laurell WS-650MZ-23NPPB spin coater equipped with a GAST 0523–101Q-G588NDX Vacuum Pump) on FTO glass. Optimal film homogeneity was obtained operating at 5000 rpm for 1 min, with either 3× 50 μL (Bi HSs).

#### Photoelectrochemical Setup

The photoelectrochemical characterization of the sample was carried out in a single compartment quartz cell, purchased from PineResearch (“Low Volume Photoelectrochemical Three Electrode Quartz Cell”). Tests were performed under a typical three-electrode configuration, using the above-described photoelectrodes as working electrode (WE), a Pt coil as counter electrode (CE) and an Ag wire (sealed in a fritted glass tube, filled with the working electrolyte) as reference electrode (RE). Gas inlets and outlets were also implemented. The whole cell was gastight, with the gas outlet connected to an inline gas chromatograph (GC). The testing electrolyte was a 0.1 M LiClO_4_ solution in ACN:H_2_O (99:1 v/v). A THORLABS M415L4–415 nm, 1310 mW (Min) Mounted LED, 1500 mA was employed as illumination source and an Ivium Compact Stat.h as the potentiostat. The tests were performed by feeding the cell by bubbling Ar (delivered by a Bronkhorst F-201CV-100-RGD-22-V mass flow controller) or CO_2_ (delivered by a Bronkhorst F-201CV-100-RGD-22-V mass flow controller), depending on the nature of the test, with a flow rate set at 5.5 sccm. The gas outlet was connected in-line to a gas dryer (SRI Gas Stream Dryer, SRI part# 8670–5850), to a universal Mass Flow Meter (EL-Flow Prestige Bronkhorst, model FG-111B-AGD-22-V-DA-000) and then to the GC for the quantification of gaseous products.

#### Electrochemical Tests

Three main electrochemical techniques were used in the characterization of the CO_2_RR photoelectroactivity of the samples under study. Open Circuit Potential (OCP) was measured under illumination and/or dark conditions to assess the interaction of the thin films with the incident radiation. Cyclic Voltammetry (CV) was also operated under both illumination and dark conditions, in the presence and absence of CO_2_, depending on the nature of the test, aiming to assess the CO_2_RR features of the materials. Finally, ChronoAmperometric scans (CA, 1 h-long) were recorded, again under both illumination and dark conditions, as to determine the CO_2_RR product distribution yielded by the materials at definite potentials. Unless otherwise stated, all the potentials reported in this work were measured versus the previously described RE. Blank tests were performed on the electrode support (i.e., bare FTO, 1 × 1 cm).

#### Product Quantification

Gaseous products were detected by an inline-connected SRI 8610C gas chromatograph (Multiple Gas Analyzer #5) equipped with a thermal conductivity detector (TCD) and flame ionization detector (FID) coupled with a methanizer. Argon was used as a gas carrier. Detectors response were calibrated prior to experiments using a customized gas mixture containing the most typical CO_2_RR gas phase products. Details on calibration and quantification methods can be found in our previous works.^59^ Liquid products were detected ex-situ, sampling the postreaction electrolytes, by HPLC. All HPLC separations were carried out on an Agilent Infinity 1260, equipped with a quaternary pump, standard autosampler, thermostated column compartment, Diode Array Detector (DAD/UV–vis) operating in the range between 190 to 950 nm, and a Refractive Index Detector (RID). In particular, possible CO_2_RR liquid products (e.g., formic acid/formate ions) were separated on an Agilent Hi-Plex H (300 × 7.7 mm) operated at 50 °C in 5 mM M H_2_SO_4(aq)_, isocratic mode, 0.6 mL min^–1^, 25 μL injection volume. Chromatographic runs lasted for 30 min, allowing for the elution of all common CO_2_RR products. Products were detected by either DAD/UV–vis (210 and 280 nm) and/or RID (*T* = 40 °C) depending on the nature of the analyte. A complete overview on the separation of liquid CO_2_RR products according to this methodology can be found in the literature.^65^

## Supporting Information Available

Supporting figures including TEM images and XRD pattern of synthesis with and without oleylamine; TEM images, XRD Rietveld fits, and size evolution of aliquots at different reaction time; HAADF image and STEM-EDX compositional map of heterostructures; expanded description of epitaxial interface identified with Ogre; UV spectra and photographs of photocatalysis experiments; table of Z potential; open circuit voltage measures, cyclic voltammetry, chronoamperometry, GC, and HPLC chromatographs of blank and heterostructures samples (PDF)

## References

[ref1] Bera S.; Pradhan N. Perovskite Nanocrystal Heterostructures: Synthesis, Optical Properties, and Applications. ACS Energy Lett. 2020, 5 (9), 2858–2872. 10.1021/acsenergylett.0c01449.

[ref2] Carbone L.; Cozzoli P. D. Colloidal Heterostructured Nanocrystals: Synthesis and Growth Mechanisms. Nano Today 2010, 5 (5), 449–493. 10.1016/j.nantod.2010.08.006.

[ref3] Liu J.; Zhang J. Nanointerface Chemistry: Lattice-Mismatch-Directed Synthesis and Application of Hybrid Nanocrystals. Chem. Rev. 2020, 120 (4), 2123–2170. 10.1021/acs.chemrev.9b00443. 31971378

[ref4] Ha M.; Kim J.-H.; You M.; Li Q.; Fan C.; Nam J.-M. Multicomponent Plasmonic Nanoparticles: From Heterostructured Nanoparticles to Colloidal Composite Nanostructures. Chem. Rev. 2019, 119 (24), 12208–12278. 10.1021/acs.chemrev.9b00234. 31794202

[ref5] Haldar K. K.; Sinha G.; Lahtinen J.; Patra A. Hybrid Colloidal Au-CdSe Pentapod Heterostructures Synthesis and Their Photocatalytic Properties. ACS Appl. Mater. Interfaces 2012, 4 (11), 6266–6272. 10.1021/am301859b. 23113704

[ref6] Wu K.; Zhu H.; Liu Z.; Rodriguez-Cordoba W.; Lian T. Ultrafast Charge Separation and Long-Lived Charge Separated State in Photocatalytic CdS–Pt Nanorod Heterostructures. J. Am. Chem. Soc. 2012, 134 (25), 10337–10340. 10.1021/ja303306u. 22655858

[ref7] Pradhan N. Design Strategies for Epitaxial Metal(0)–Halide Perovskite Nanocrystal Heterostructures. ACS Energy Lett. 2024, 9 (5), 2378–2386. 10.1021/acsenergylett.4c00855.

[ref8] Milliron D. J.; Hughes S. M.; Cui Y.; Manna L.; Li J.; Wang L.-W.; Paul Alivisatos A. Colloidal Nanocrystal Heterostructures with Linear and Branched Topology. Nature 2004, 430 (6996), 190–195. 10.1038/nature02695. 15241410

[ref9] Huynh W. U.; Dittmer J. J.; Alivisatos A. P. Hybrid Nanorod-Polymer Solar Cells. Science 2002, 295 (5564), 2425–2427. 10.1126/science.1069156. 11923531

[ref10] Shim M. Colloidal Nanorod Heterostructures for Photovoltaics and Optoelectronics. J. Phys. D: Appl. Phys. 2017, 50 (17), 173002 10.1088/1361-6463/aa65a5.

[ref11] Luo J.; Selopal G. S.; Tong X.; Wang Z. Colloidal Quantum Dots and Two-dimensional Material Heterostructures for Photodetector Applications. Electron 2024, 2 (2), e30 10.1002/elt2.30.

[ref12] Vaneski A.; Susha A. S.; Rodriguez-Fernandez J.; Berr M.; Jackel F.; Feldmann J.; Rogach A. L. Hybrid Colloidal Heterostructures of Anisotropic Semiconductor Nanocrystals Decorated with Noble Metals: Synthesis and Function. Adv. Funct. Mater. 2011, 21 (9), 1547–1556. 10.1002/adfm.201002444.

[ref13] Das R.; Patra A.; Dutta S. K.; Shyamal S.; Pradhan N. Facets-Directed Epitaxially Grown Lead Halide Perovskite-Sulfobromide Nanocrystal Heterostructures and Their Improved Photocatalytic Activity. J. Am. Chem. Soc. 2022, 144 (40), 18629–18641. 10.1021/jacs.2c08639. 36174102

[ref14] Razgoniaeva N.; Moroz P.; Lambright S.; Zamkov M.; et al. Photocatalytic Applications of Colloidal Heterostructured Nanocrystals: What’s Next?. J. Phys. Chem. Lett. 2015, 6 (21), 4352–4359. 10.1021/acs.jpclett.5b01883. 26722971

[ref15] Palazon F. Metal Chalcohalides: Next Generation Photovoltaic Materials?. Solar RRL 2022, 6 (2), 2100829 10.1002/solr.202100829. PMC7613291 35966398

[ref16] Ghorpade U. V.; Suryawanshi M. P.; Green M. A.; Wu T.; Hao X.; Ryan K. M.; et al. Emerging Chalcohalide Materials for Energy Applications. Chem. Rev. 2023, 123 (1), 327–378. 10.1021/acs.chemrev.2c00422. 36410039 PMC9837823

[ref17] Li J.; Han S.-S.; Guo S.-P. Chalcohalides: A Rising Type of Second-Order Nonlinear Optical Materials. Eur. J. Inorg. Chem. 2022, 2022 (33), e202200419 10.1002/ejic.202200419.

[ref18] Toso S.; Akkerman Q. A.; Martin-Garcia B.; Prato M.; Zito J.; Infante I.; Dang Z.; Moliterni A.; Giannini C.; Bladt E.; et al. Nanocrystals of Lead Chalcohalides: A Series of Kinetically Trapped Metastable Nanostructures. J. Am. Chem. Soc. 2020, 142 (22), 10198–10211. 10.1021/jacs.0c03577. 32374173 PMC7737912

[ref19] Quarta D.; Toso S.; Giannuzzi R.; Caliandro R.; Moliterni A.; Saleh G.; Capodilupo A.-L.; Debellis D.; Prato M.; Nobile C.; et al. Colloidal Bismuth Chalcohalide Nanocrystals. Angew. Chem. Int. Ed. 2022, 61, e202201747 10.1002/ange.202201747. PMC9311208 35226780

[ref20] He J.; Hu X.; Liu Z.; Chen W.; Longo G. Prospect for Bismuth/Antimony Chalcohalides-Based Solar Cells. Adv. Funct. Mater. 2023, 33 (48), 2306075 10.1002/adfm.202306075.

[ref21] Choi Y. C.; Jung K.-W. Recent Progress in Fabrication of Antimony/Bismuth Chalcohalides for Lead-Free Solar Cell Applications. Nanomater. 2020, 10 (11), 2284 10.3390/nano10112284. PMC7698906 33218079

[ref22] Quarta D.; Toso S.; Fieramosca A.; Dominici L.; Caliandro R.; Moliterni A.; Tobaldi D. M.; Saleh G.; Gushchina I.; Brescia R.; et al. Direct Band Gap Chalcohalide Semiconductors: Quaternary AgBiSCl_2_ Nanocrystals. Chem. Mater. 2023, 35 (23), 9900–9906. 10.1021/acs.chemmater.3c01403.

[ref23] Kunioku H.; Higashi M.; Abe R. Low-Temperature Synthesis of Bismuth Chalcohalides: Candidate Photovoltaic Materialswith Easily, Continuously Controllable Band Gap. Sci. Rep. 2016, 6 (1), 32664 10.1038/srep32664. 27600662 PMC5013401

[ref24] Govindaraj P.; Venugopal K. Intrinsic Ultra-Low Lattice Thermal Conductivity in Orthorhombic BiSI: An Excellent Thermoelectric Material. J. Alloys Compd. 2022, 929, 167347 10.1016/j.jallcom.2022.167347.

[ref25] Govindaraj P.; Murugan K.; Venugopal K. Anisotropic Electron and Phonon Transport Properties in Pnictogen Chalcohalides: PnSI (Pn = Sb, Bi). ACS Appl. Energy Mater. 2023, 6 (20), 10639–10651. 10.1021/acsaem.3c01811.

[ref26] Ghorpade U. V.; Suryawanshi M. P.; Green M. A.; Wu T.; Hao X.; Ryan K. M.; et al. Emerging Chalcohalide Materials for Energy Applications. Chem. Rev. 2023, 123 (1), 327–378. 10.1021/acs.chemrev.2c00422. 36410039 PMC9837823

[ref27] Shyamal S.; Pradhan N. Nanostructured Metal Chalcohalide Photocatalysts: Crystal Structures, Synthesis, and Applications. ACS Energy Lett. 2023, 8 (9), 3902–3926. 10.1021/acsenergylett.3c01236.

[ref28] Wang L.; Hung Y.-C.; Hwu S.-J.; Koo H.-J.; Whangbo M.-H.; et al. Synthesis, Structure, and Properties of a New Family of Mixed-Framework Chalcohalide Semiconductors: CdSbS_2_X (X = Cl, Br), CdBiS_2_X (X = Cl, Br), and CdBiSe_2_X (X = Br, I). Chem. Mater. 2006, 18 (5), 1219–1225. 10.1021/cm0522230.

[ref30] Quarta D.; Toso S.; Saleh G.; Caliandro R.; Moliterni A.; Griesi A.; Divitini G.; Infante I.; Gigli G.; Giannini C.; et al. Mixed Valence of Bismuth in Hexagonal Chalcohalide Nanocrystals. Chem. Mater. 2023, 35 (3), 1029–1036. 10.1021/acs.chemmater.2c02941.

[ref31] Rusch P.; Toso S.; Ivanov Y. P.; Marras S.; Divitini G.; Manna L. Nanocrystal Heterostructures Based On Halide Perovskites and Lead–Bismuth Chalcogenides. Chem. Mater. 2023, 35 (24), 10684–10693. 10.1021/acs.chemmater.3c02503.

[ref32] Patra A.; Jagadish K.; Shyamal S.; Ravishankar N.; Pradhan N. Perovskite-Chalcogenide Epitaxial Heterostructures: Possibility of Multiple Axial Orientations of CsPbBr_3_ Nanocrystals on PbBi_2_S_4_ Nanorods. Chem. Mater. 2024, 36 (8), 3803–3811. 10.1021/acs.chemmater.4c00124.

[ref35] Groom R.; Jacobs A.; Cepeda M.; Drummey R.; Latturner S. E. Bi_13_S_18_I_2_ : (Re)Discovery of a Subvalent Bismuth Compound Featuring [Bi_2_ ]^4+^ Dimers Grown in Sulfur/Iodine Flux Mixtures. Chem. Mater. 2017, 29 (7), 3314–3323. 10.1021/acs.chemmater.7b00702.

[ref36] Das A.; Debnath K.; Maria I.; Das S.; Dutta P.; Swain D.; Waghmare U. V.; Biswas K.; et al. Influence of Subvalent Twin-Rattler for High *n* -Type Thermoelectric Performance in Bi_13_S_18_Br_2_ Chalcohalide. J. Am. Chem. Soc. 2024, 146 (44), 30518–30528. 10.1021/jacs.4c11738. 39449606

[ref37] Bergmann J.; Monecke T.; Kleeberg R. Alternative Algorithm for the Correction of Preferred Orientation in Rietveld Analysis. J. Appl. Crystallogr. 2001, 34 (1), 16–19. 10.1107/S002188980001623X.

[ref38] Alemayehu A.; Zakutna D.; Kohutekova S.; Tyrpekl V. Transition between Two Solid-solutions: Effective and Easy Way for Fine Ce_1–x_Gd_x_O_2–x/2_ Powders Preparation. J. Am. Ceram. Soc. 2022, 105, 4621–4631. 10.1111/jace.18443.

[ref40] Rodriguez-Carvajal J. Recent Advances in Magnetic Structure Determination by Neutron Powder Diffraction. Phys. B: Condens. Matter 1993, 192 (1), 55–69. 10.1016/0921-4526(93)90108-I.

[ref42] Toso S.; Dardzinski D.; Manna L.; Marom N. Structure Prediction of Ionic Epitaxial Interfaces with Ogre Demonstrated for Colloidal Heterostructures of Lead Halide Perovskites. ACS Nano 2025, 19, 5326–5341. 10.1021/acsnano.4c12713. 39893669 PMC11823643

[ref43] Zhou C.; Zheng K.; Lu Z.; Zhang Z.; Liao Z.; Chen P.; Lu W.; Zou J. Quality Control of GaAs Nanowire Structures by Limiting As Flux in Molecular Beam Epitaxy. J. Phys. Chem. C 2015, 119 (35), 20721–20727. 10.1021/acs.jpcc.5b05606.

[ref44] Leshchenko E. D.; Sibirev N. V. Recent Advances in the Growth and Compositional Modelling of III–V Nanowire Heterostructures. Nanomater. 2024, 14 (22), 1816 10.3390/nano14221816. PMC11597288 39591057

[ref45] Johansson J.; Dick K. A. Recent Advances in Semiconductor Nanowire Heterostructures. CrystEngComm 2011, 13 (24), 7175–7184. 10.1039/c1ce05821e.

[ref46] Zhang H.; Delikanli S.; Qin Y.; He S.; Swihart M.; Zeng H. Synthesis of Monodisperse CdS Nanorods Catalyzed by Au Nanoparticles. Nano Res. 2008, 1 (4), 314–320. 10.1007/s12274-008-8032-5.

[ref47] Savitzky B. H.; Zeltmann S. E.; Hughes L. A.; Brown H. G.; Zhao S.; Pelz P. M.; Pekin T. C.; Barnard E. S.; Donohue J.; Rangel DaCosta L.; et al. py4DSTEM: A Software Package for Four-Dimensional Scanning Transmission Electron Microscopy Data Analysis. Micros. Microanal. 2021, 27 (4), 712–743. 10.1017/S1431927621000477. 34018475

[ref48] Ai L.; Jia D.; Guo N.; Xu M.; Zhang S.; Wang L.; Jia L.; et al. Controlled Growth of Single-Crystalline Bi_.333_(Bi_6_S_9_)Br Nanorods under Hydrothermal Conditions for Enhanced Photocatalytic Reduction of Cr (VI). J. Alloys Compd. 2020, 842, 155879 10.1016/j.jallcom.2020.155879.

[ref50] Nguyen V. H.; Lee T.; Nguyen T. D. Solvothermal Synthesis of Bismuth-Based Halide Perovskite Nanostructures for Photocatalytic Degradation of Organic Pollutants under LED Light Irradiation. ACS Appl. Nano Mater. 2023, 6 (5), 3435–3445. 10.1021/acsanm.2c05218.

[ref51] Yu Yu; et al. A Bi/BiOCl Heterojunction Photocatalyst with Enhanced Electron–Hole Separation and Excellent Visible Light Photodegrading Activity. J. Mater. Chem. A 2014, 2 (6), 1677–81. 10.1039/C3TA14494A.

[ref52] Fonagy O.; Szabo-Bardos E.; Horvath O. 1,4-Benzoquinone and 1,4-Hydroquinone Based Determination of Electron and Superoxide Radical Formed in Heterogeneous Photocatalytic Systems. J. Photochem. Photobiol., A 2021, 407, 113057 10.1016/j.jphotochem.2020.113057.

[ref53] Nguyen T. T. D.; Nguyen D.; Vo P. P.; Doan H. N.; Pham H. T. N.; Hoang V. H.; Tien Le K.; Kinashi K.; Huynh V. T.; Nguyen P. T. The Roles of Ethanol and Isopropanol as Hole Scavengers in the Photoreduction Reaction of Graphene Oxide by TiO_2_: A Competition of Oxygenated Groups Removal and Carbon Defects Invasion. J. Mol. Liq. 2023, 381, 121831 10.1016/j.molliq.2023.121831.

[ref54] Gracien E. B.; Jeremie M. L.; Joseph L. K.-K.; Omer M. M.; Antoine M. K.; Hercule K. M.; Gerard M. N. Role of Hydroxyl Radical Scavenger Agents in Preparing Silver Nanoparticles under γ-Irradiation. SN Appl. Sci. 2019, 1 (9), 961 10.1007/s42452-019-0973-7.

[ref55] Zhao X.; Chen H.; Chen X.; Hu J.; Wu T.; Wu L.; Li M. Multiple Halide Anion Doped Layered Bismuth Terephthalate with Excellent Photocatalysis for Pollutant Removal. RSC Adv. 2018, 8 (67), 38370–38375. 10.1039/C8RA08493A. 35559113 PMC9089753

[ref56] Gao F.; Zhao Y.; Li Y.; Wu G.; Lu Y.; Song Y.; Huang Z.; Li N.; Zhao J. Hierarchical Bi Based Nanobundles: An Excellent Photocatalyst for Visible-Light Degradation of Rhodamine B Dye. J. Colloid Interface Sci. 2015, 448, 564–572. 10.1016/j.jcis.2015.02.056. 25792479

[ref57] Percivalle N. M.; Carofiglio M.; Hernandez S.; Cauda V. Ultra-Fast Photocatalytic Degradation of Rhodamine B Exploiting Oleate-Stabilized Zinc Oxide Nanoparticles. Discover Nano 2024, 19 (1), 126 10.1186/s11671-024-04077-7. 39120807 PMC11315820

[ref58] Denisov N.; Yoo J.; Schmuki P. Effect of Different Hole Scavengers on the Photoelectrochemical Properties and Photocatalytic Hydrogen Evolution Performance of Pristine and Pt-Decorated TiO_2_ Nanotubes. Electrochim. Acta 2019, 319, 61–71. 10.1016/j.electacta.2019.06.173.

[ref59] Bellato F.; Ferri M.; Zhu D.; Le T.-H.-H.; Annamalai A.; Rizzo M.; Martin I.; Goldoni L.; Brescia R.; Prato M.; et al. Indium Arsenide Quantum Dot Derived Catalyst for Selective CO_2_ Electrochemical Reduction to Formate. ACS Energy Lett. 2024, 9 (3), 1097–1102. 10.1021/acsenergylett.4c00295.

[ref60] Elgrishi N.; Rountree K. J.; McCarthy B. D.; Rountree E. S.; Eisenhart T. T.; Dempsey J. L. A Practical Beginner’s Guide to Cyclic Voltammetry. J. Chem. Educ. 2018, 95 (2), 197–206. 10.1021/acs.jchemed.7b00361.

[ref65] Iglesias van Montfort H.-P.; Subramanian S.; Irtem E.; Sassenburg M.; Li M.; Kok J.; Middelkoop J.; Burdyny T.; et al. An Advanced Guide to Assembly and Operation of CO_2_ Electrolyzers. ACS Energy Lett. 2023, 8 (10), 4156–4161. 10.1021/acsenergylett.3c01561. PMC9841604 36660371

