# Supplementary material for: Synthesis, Growth Mechanism, and Photocatalytic Properties of Metallic-Bi/Bi13S18Br2 Nano-Bell Heterostructures
Source: ACS Mater Lett. 2025 Apr 1;7(5):1707–16. doi: 10.1021/acsmaterialslett.5c00043 (PMC12056758; doi:10.1021/acsmaterialslett.5c00043)
Supplement: Supplementary file 1 — tz5c00043_si_001.pdf [file tz5c00043_si_001.pdf]

Synthesis, Growth Mechanism, and Photocatalytic Properties of Metallic-Bi/$Bi_{13}S_{18}Br_2$ Nano-Bell Heterostructures

Anna Cabona[1,2], Stefano Toso[1,3], Andrea Griesi[4], Martina Rizzo[1,2], Michele Ferri[1], Pascal Rusch[1], Giorgio Divitini[4], Julia Pérez-Prieto*[5], Raquel E. Galian*[5], Ilka Kriegel*[2], Liberato Manna*[1]

[1] Nanochemistry Department, Italian Institute of Technology, Via Morego 30, 16163 Genova, Italy
[2] Department of Applied Science and Technology, Politecnico di Torino, Corso Duca degli Abruzzi 34, 10129 Turin, Italy
[3] Lund University, Division of Chemical Physics, Naturvetarvägen 14, 221 00 Lund, Sweden
[4] Electron Spectroscopy and Nanoscopy, Italian Institute of Technology, Via Morego 30, 16163 Genoa, Italy
[5] Institute of Molecular Science, University of Valencia, c/Catedrático José Beltrán Martínez 2, 46980 Paterna, Valencia, Spain

SUPPORTING INFORMATION

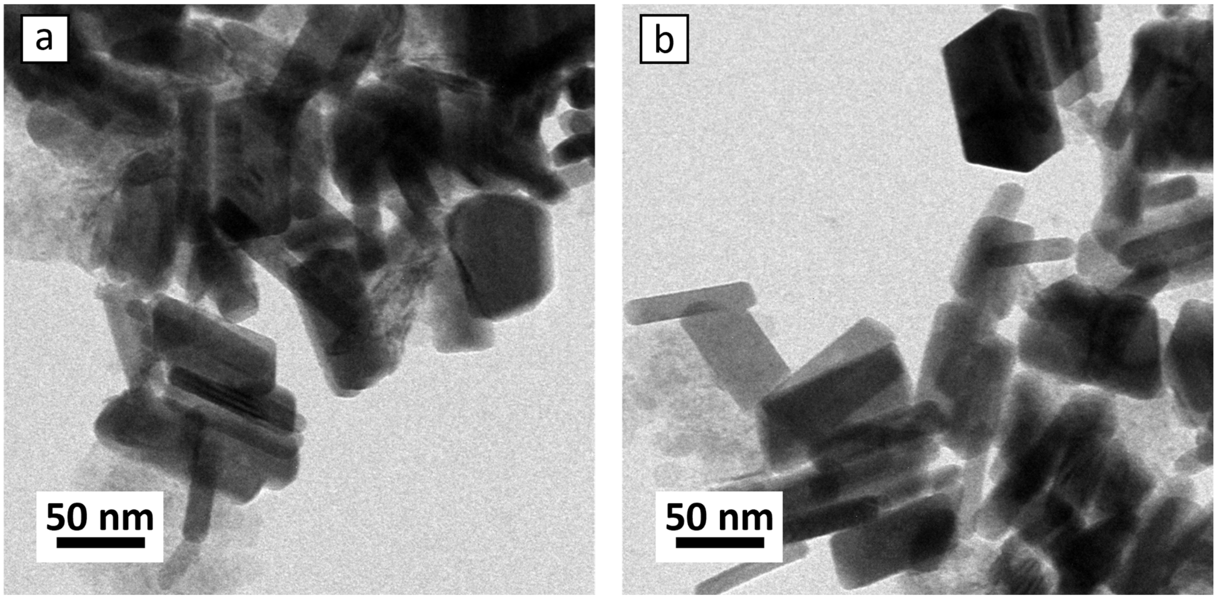

Figure S1. Control synthesis in the absence of amines. a-b) TEM images of colloidal BiSBr NCs synthetized in the absence of amines, following the procedure reported by Quarta et al.[i]

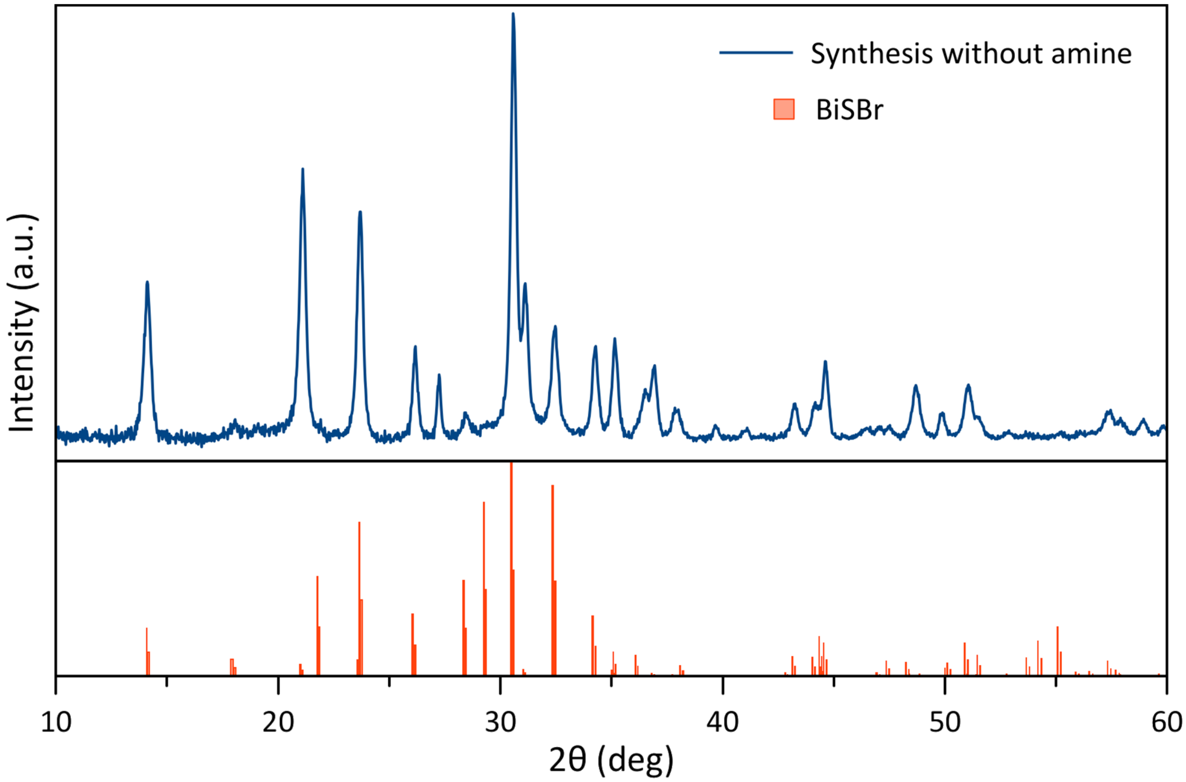

Figure S2. Control synthesis in the absence of amines. XRD pattern of colloidal BiSBr NCs synthetized in the absence of amine, confirming the phase attribution to BiSBr.

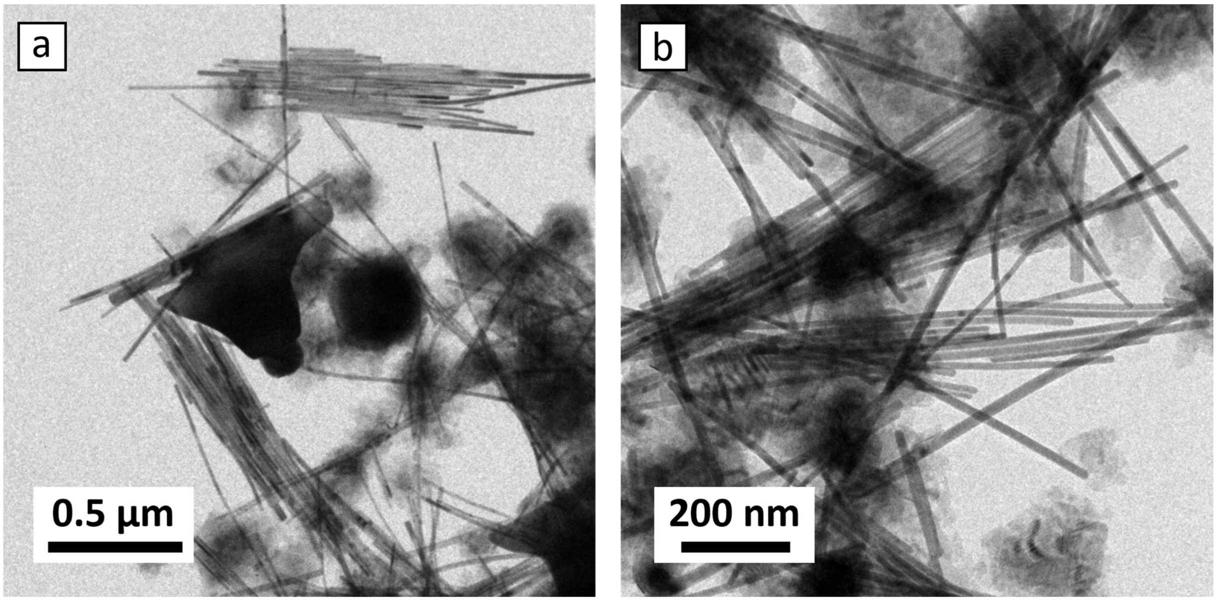

Figure S3. Synthesis performed with added oleylamine. TEM images of colloidal $Bi_{13}S_{18}Br_2$ obtained by adding 220 µL of oleylamine on top of the reagents used for Figures S1-2 while adopting identical reaction conditions.

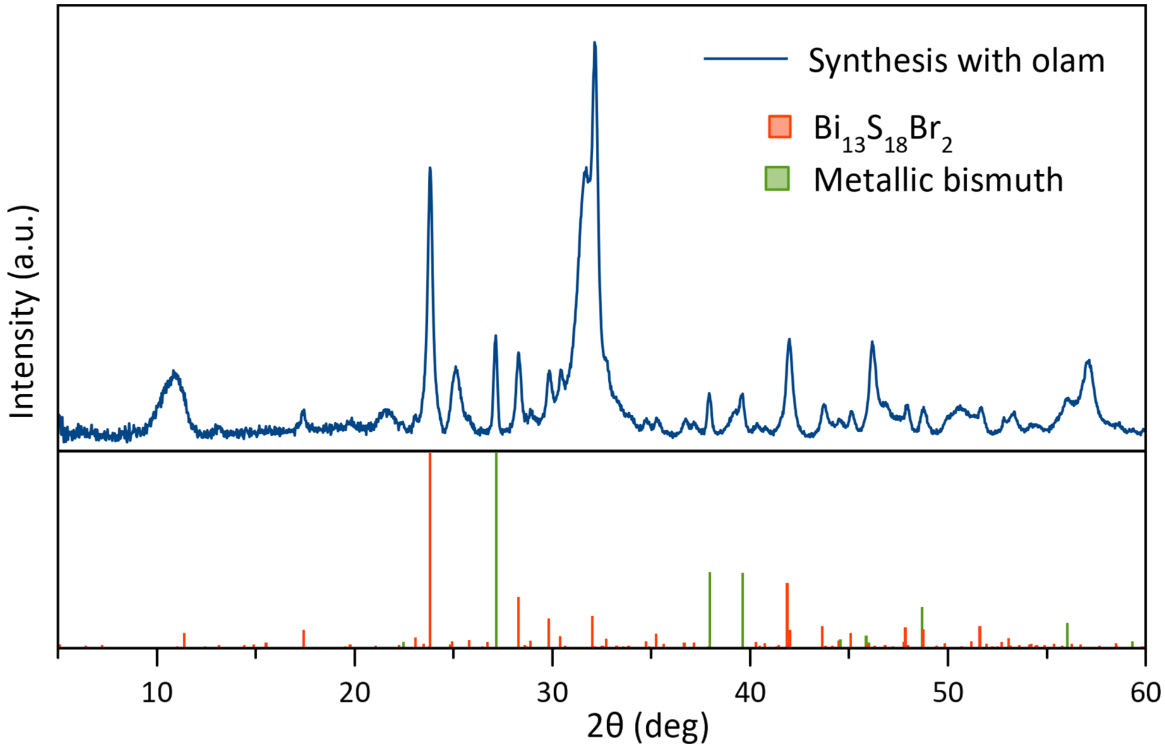

Figure S4. Synthesis performed with added oleylamine. XRD pattern of the colloidal $Bi_{13}S_{18}Br_2$ nanorods obtained by adding oleylamine (see Figure S3).

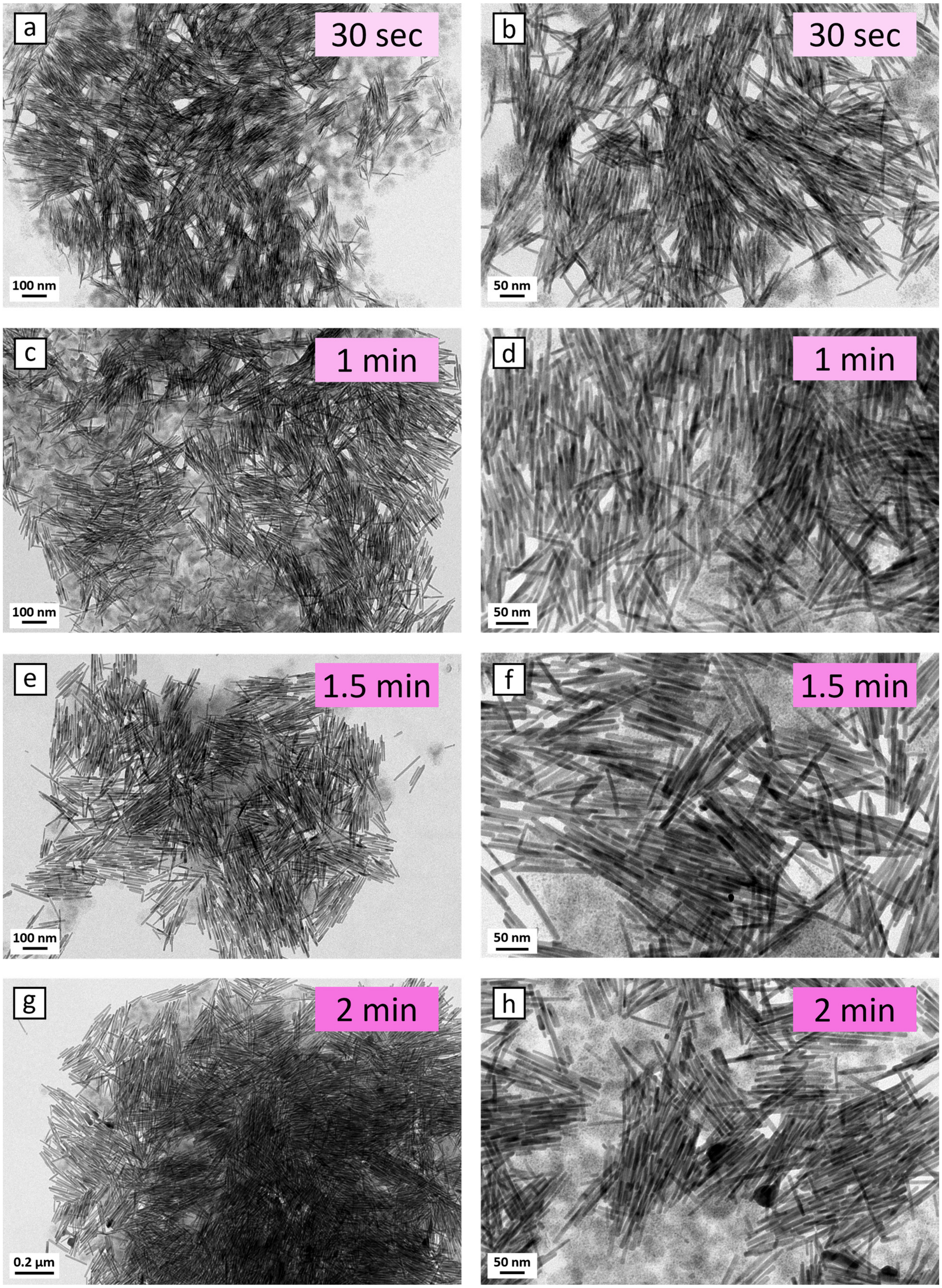

Figure S5. Morphology evolution. TEM images of $Bi/Bi_{13}S_{18}Br_2$ solution aliquots taken at 30 s (a-b), 60 s (c-d), 1.5 min (e-f), and 2 min (g-h) after the start of the reaction.

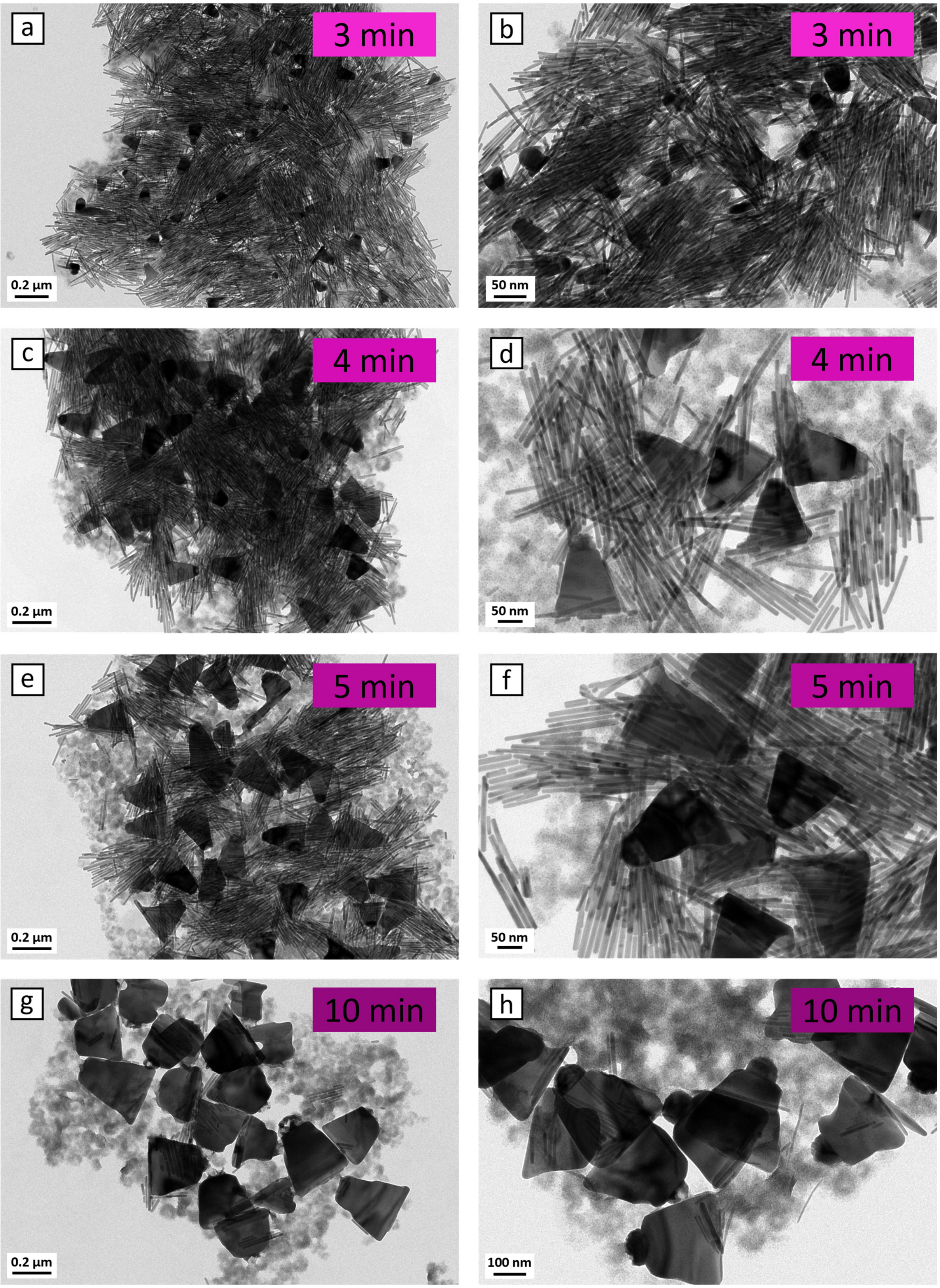

Figure S6. Morphology evolution (continued). TEM images of $Bi/Bi_{13}S_{18}Br_2$ solution aliquots taken at 3 min (a-b), 4 min (c-d), 5 min (e-f), and 10 min (g-h) after the start of the reaction.

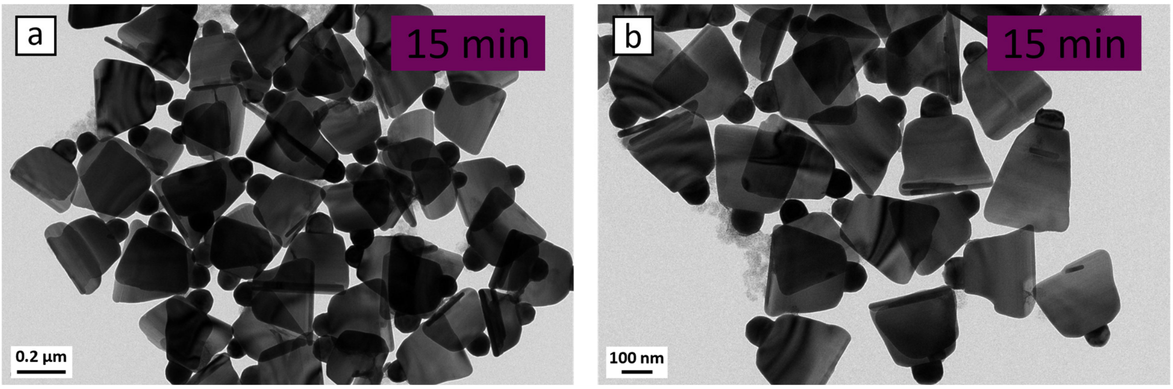

Figure S7. Morphology evolution (continued). TEM images of Bi/$Bi_{13}S_{18}Br_2$ solution aliquots taken at 15 min (a-b) after the start of the reaction.

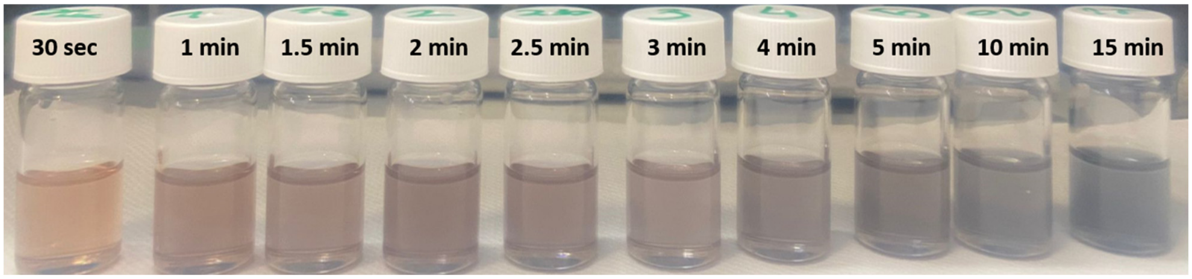

Figure S8. Aliquot color evolution. Color of the reaction medium aliquots as the reaction progresses. The solutions were diluted 1:75 to enhance the visibility of these color changes and to prepare TEM grids with well-separated particles.

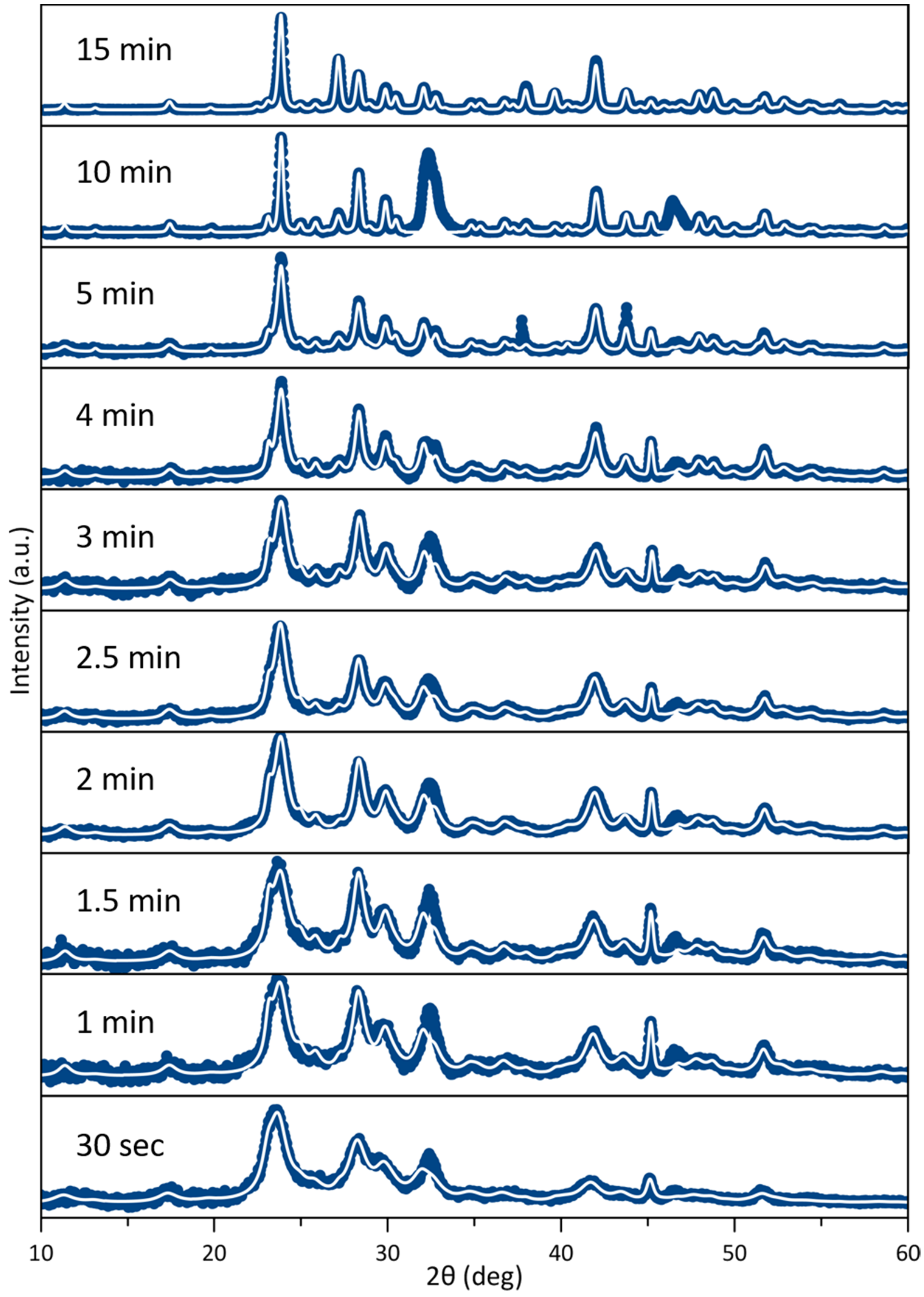

Figure S9. XRD Rietveld Fits. Rietveld fits of the XRD patterns collected from different reaction batch aliquots at various reaction times. Experimental patterns are shown in blue, while the fit traces are represented in white. To accurately capture the average dimensions of crystallites, we opted to exclude certain peaks (32.4° and 46.7°) from the fitting process when their significant preferential orientation and anisotropic broadening made it challenging to model their profiles accurately. This approach ensured that the fitting model reliably represented the remaining portions of the patterns.

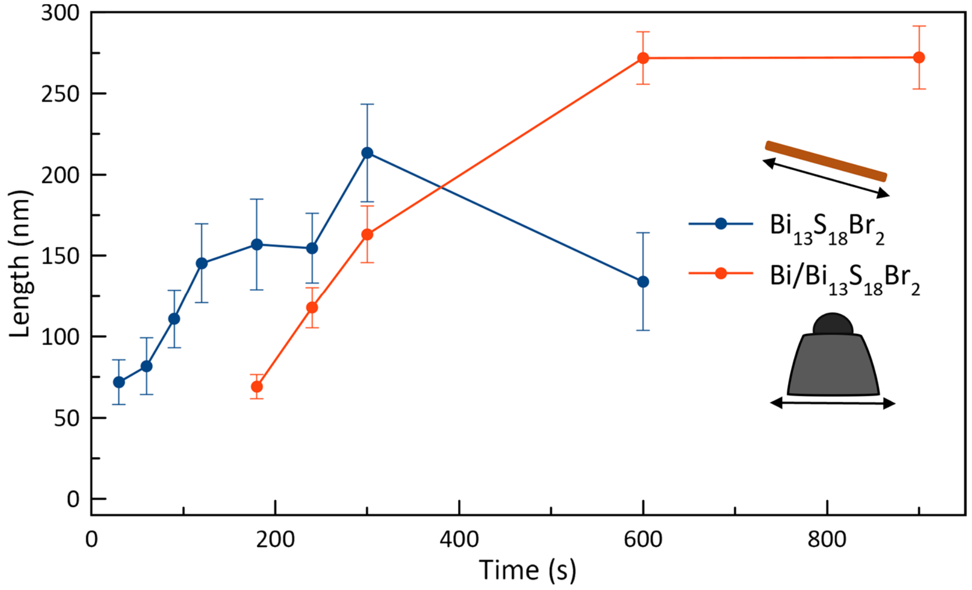

Figure S10. Size evolution of $Bi_{13}S_{18}Br_2$ by TEM. The length of free-standing $Bi_{13}S_{18}Br_2$ nanorods and of the $Bi_{13}S_{18}Br_2$ domains in $Bi/Bi_{13}S_{18}Br_2$ heterostructures was tracked by measuring 30 particles per time aliquot. Note note that there were not enough Bi/ $Bi_{13}S_{18}Br_2$ heterostructures to obtain reliable statistics before the 200-seconds mark.

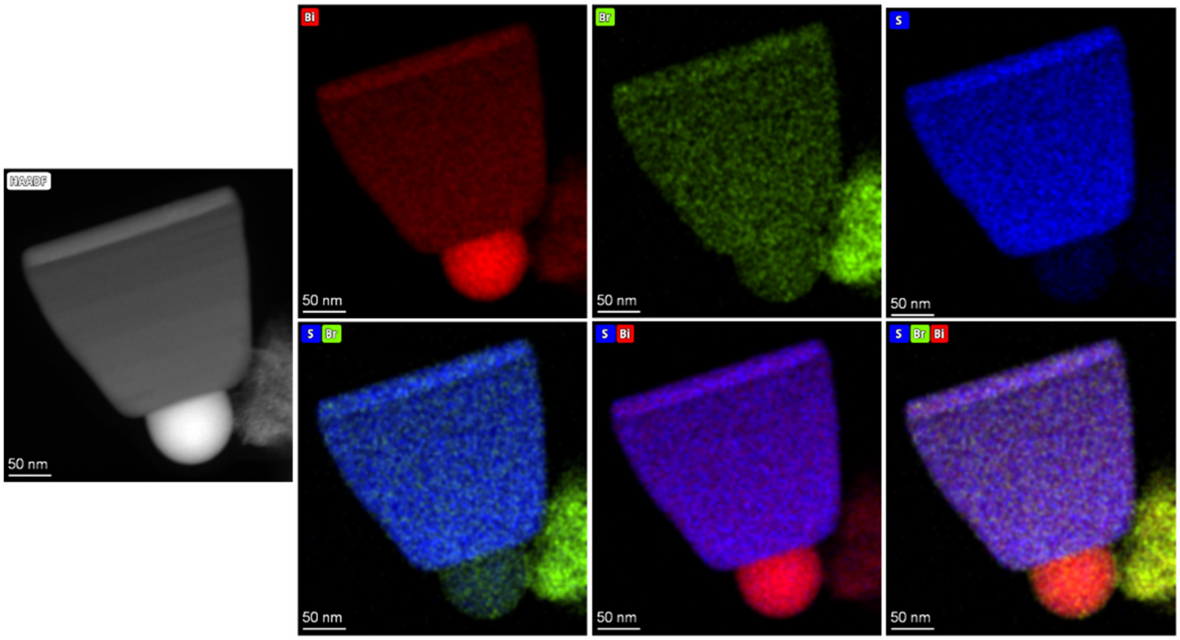

Figure S11: HAADF image and STEM-EDX compositional map of an individual nanobell, showing the presence of Bi, S and Br atoms.

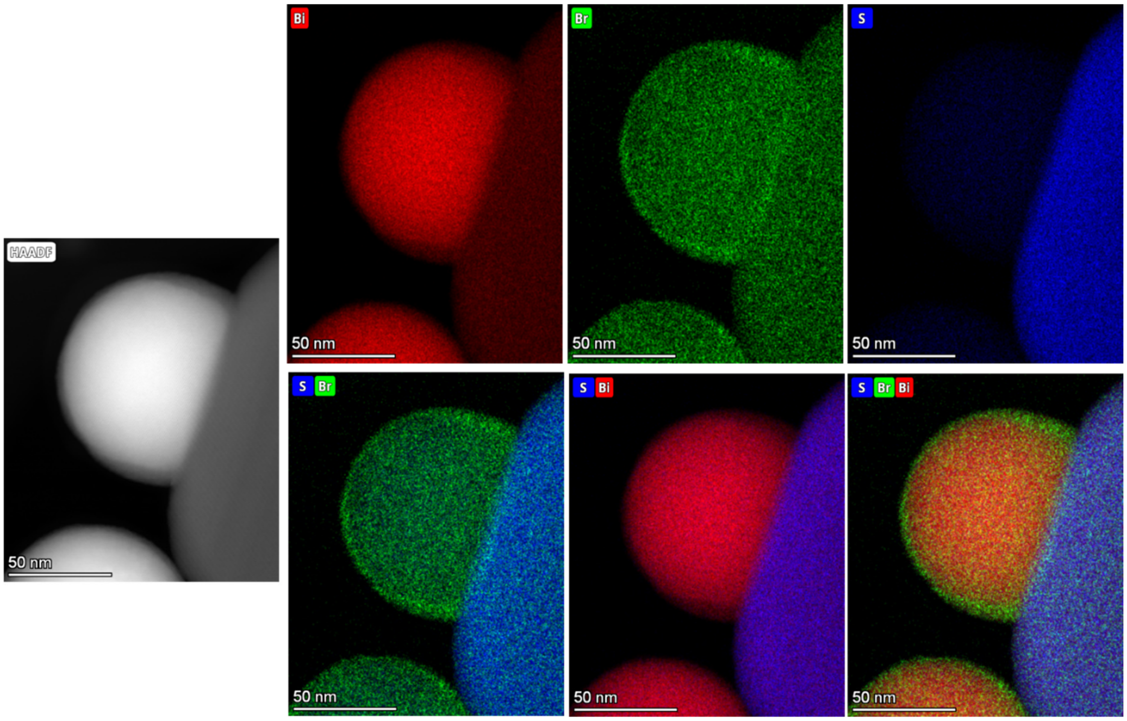

Figure S12: HAADF image and STEM-EDX compositional map of the Bi/chalcohalide contact region, showing the presence of a Br-rich shell surrounding the metal hemisphere.

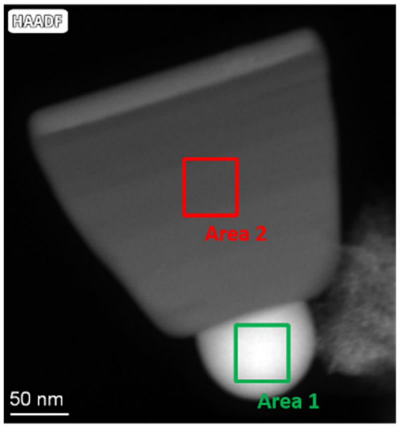

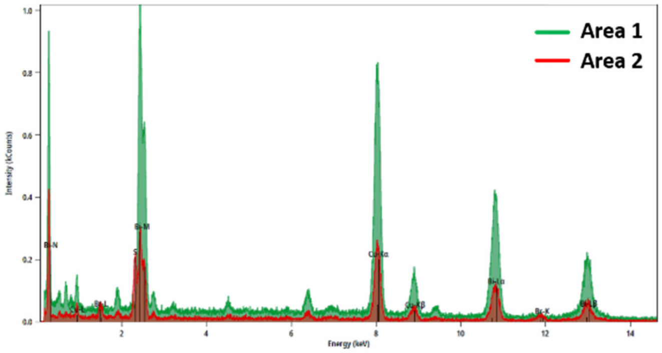

Area 1

Element	Atomic Fraction	Atomic Error	Mass Fraction	Mass Error
S	2.11	0.24	0.33	0.04
Br	0.66	0.13	0.26	0.05
Bi	97.24	0.33	99.41	0.08

Area 2

Element	Atomic Fraction	Atomic Error	Mass Fraction	Mass Error
S	47.44	2.40	13.44	1.22
Br	9.19	1.17	6.49	0.98
Bi	43.37	2.82	80.07	1.92

Figure S13: HAADF image of an individual nanobell and Energy-dispersive X-ray (EDX) analysis.

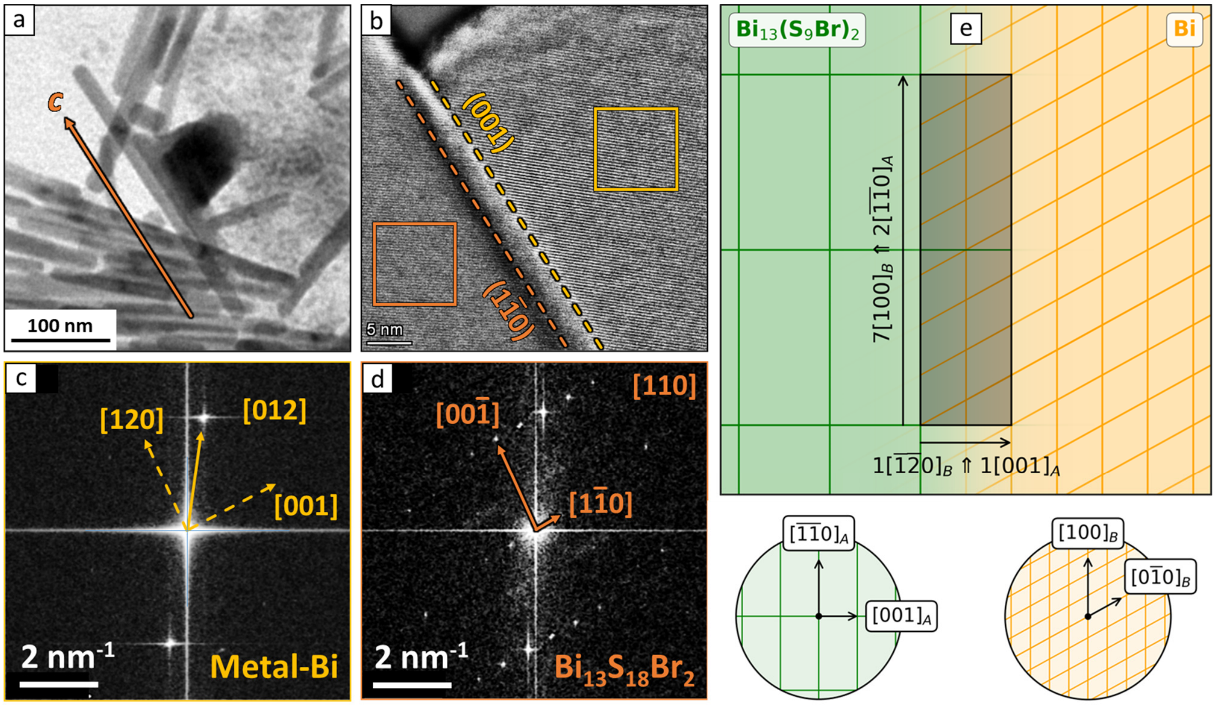

Figure S14. Identification of the 001//1-10 – Bi/$Bi_{13}S_{18}Br_2$ epitaxial relation. a) The morphology of early-stage heterostructures suggests that metallic Bi attaches to the sides of chalcohalide rods, which grow preferentially along the [001] direction. This implies that $Bi_{13}S_{18}Br_2$ exposes a (hk0) plane at the interface. b-d) Fourier transforms of HAADF images (b) support this hypothesis, as indicated by the three parallel stripes of reflections corresponding to the [hk-1], [hk0], and [hk1] planes in reciprocal space (d). The Bi orientation, however, could not be fully reconstructed from FFT due to the visibility of only a single reflection, identified as [012] based on its real-space periodicity (d = 3.28 Å). To resolve this, we used the Ogre library's lattice matching algorithm[ii] to screen potential (hkl)//(1-10) Bi/$Bi_{13}S_{18}Br_2$ interfaces (with Bi-h, k, l < 2). The epitaxial relation was evaluated by its ability to reproduce the observed relative orientation of the lattice vectors [001]-$Bi_{13}S_{18}Br_2$ and [012]-Bi. The analysis identified the (001)//(1-10) Bi/$Bi_{13}S_{18}Br_2$ relation as the most suitable, characterized by a 2D supercell with an area of 248 Å^2 and 2.4% strain, as illustrated in panel (e). The dashed lines in panel (c) mark the orientations of the Bismuth lattice vectors as predicted by Ogre. For details on the lattice matching procedure and supercell interpretation, see Ref.II.

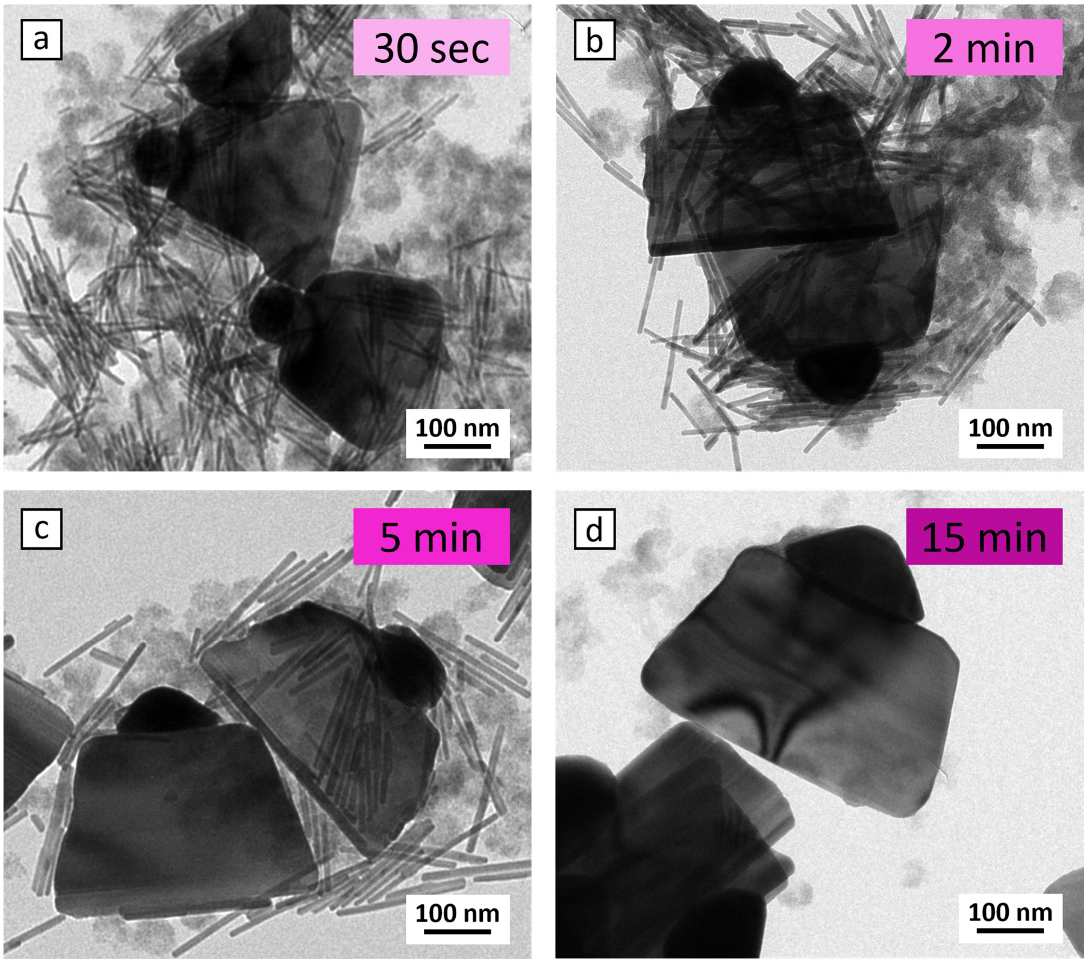

Figure 15. Ostwald ripening growth test. TEM images of Bi/$Bi_{13}S_{18}Br_2$ solution aliquots taken at a) 30 sec, b) 2 min, c) 5 min and d) 15 min after the two crude reaction solutions (one of nanorods and one of bells) have been put to react together at 180°C. The morphology evolution highlights the transfer of material from the nanorods to the nano-bells, which confirms the hypothesis of an Ostwald ripening-mediated growth mechanism. Interestingly, it is also evident how the bismuth domain becomes progressively faceted, and how at some point the chalcohalide resumes expanding along the *c*-preferred orientation growth tipical of the $Bi_{13}S_{18}Br_2$ phase.

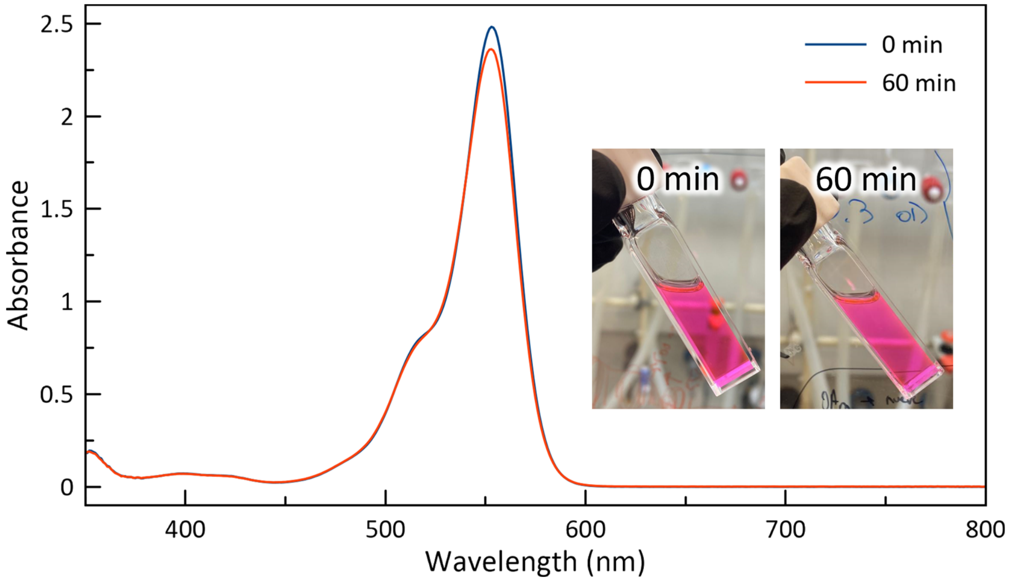

Figure S16. Rhodamine-B photostability test in the absence of photocatalyst. Absorbance spectra of the RhB solution before and after 1h of illumination at 420 nm in the absence of the photocatalyst.

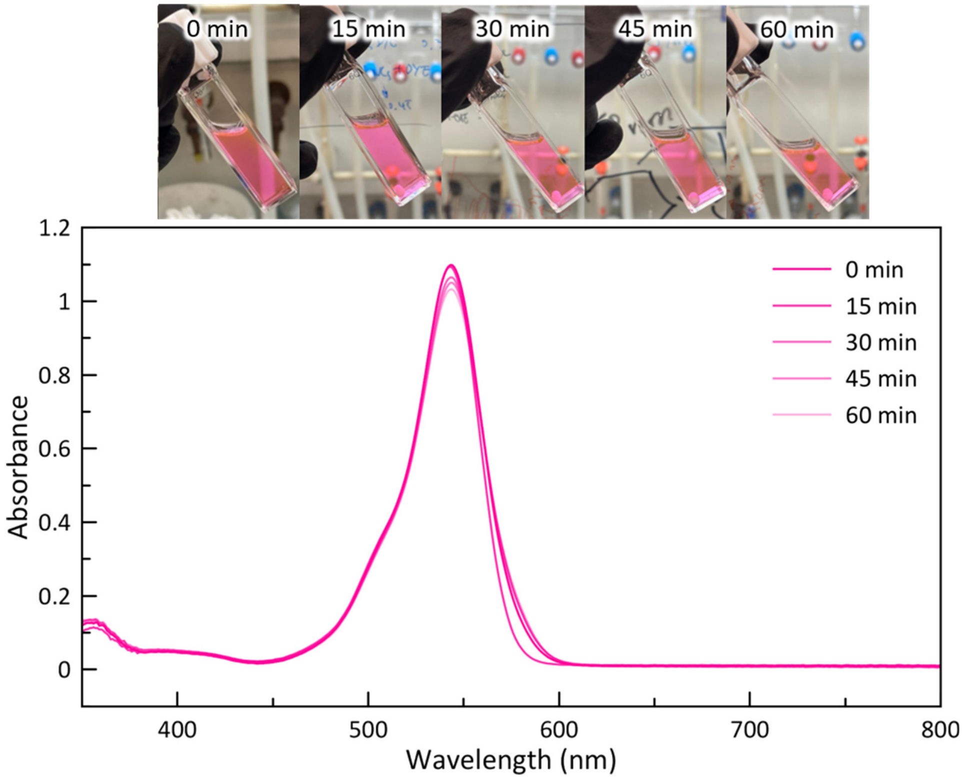

Figure S17. Rhodamine-B test in dark condition. Cuvettes images during the reaction time in dark condition and absorbance spectra of the RhB during the reaction time in dark condition, the photocatalyst was removed by centrifugation.

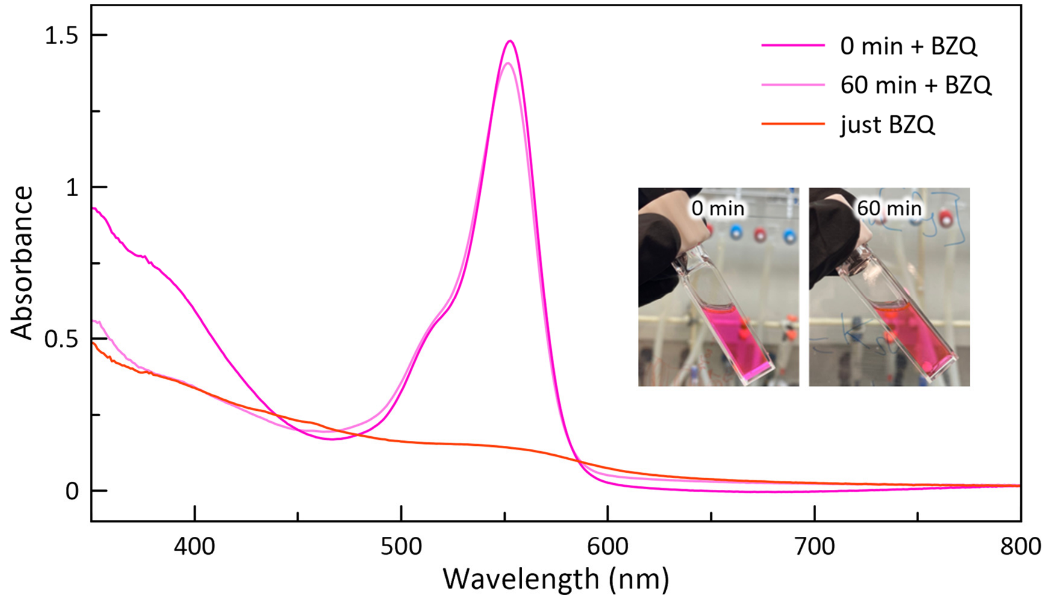

Figure S18. Rhodamine-B test in the presence of benzoquinone (BZQ). Cuvettes pictures before and after illumination with benzoquinone and absorbance spectra of RhB before and after illumination in the presence of benzoquinone, the photocatalyst was removed by centrifugation.

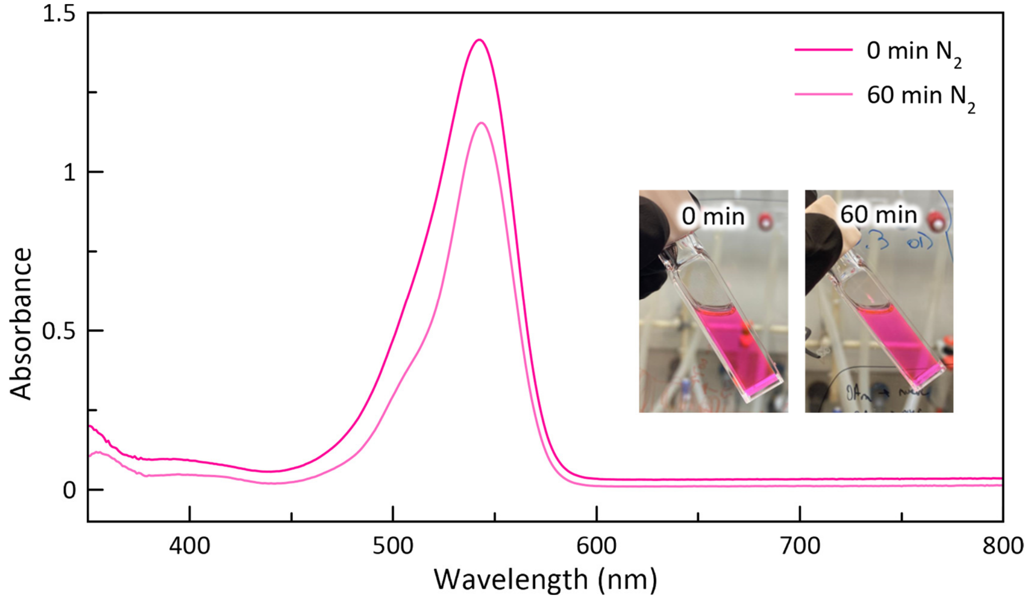

Figure S19. Rhodamine-B test in inert atmosphere (N_2). Cuvettes pictures before and after illumination in N_2 and absorbance spectra of RhB before and after illumination in N_2, the photocatalyst was removed by centrifugation.

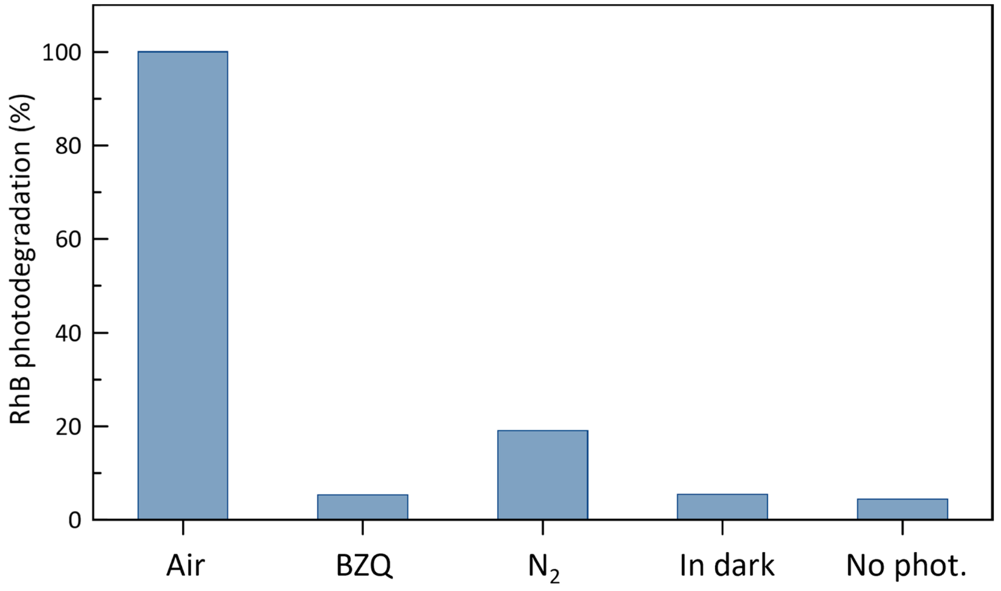

Figure S20. RhB photodegradation percentage in different conditions. Photodegradation activity of Bi/ $Bi_{13}S_{18}Br_2$ HSs in air condition, in the presence of benzoquinone (BZQ), in nitrogen atmosphere (N_2), in dark condition and in the absence of the photocatalyst (no phot.)

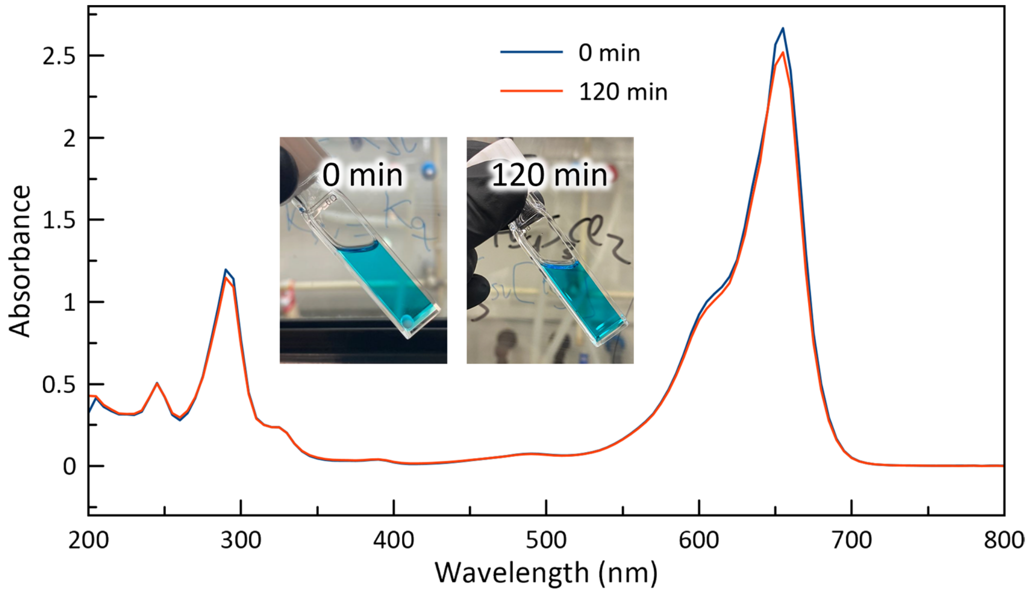

Figure S21. Methylene Blue photostability test in the absence of photocatalyst. Absorbance spectra of the MB solution before and after 2h of illumination at 420 nm in the absence of the photocatalyst.

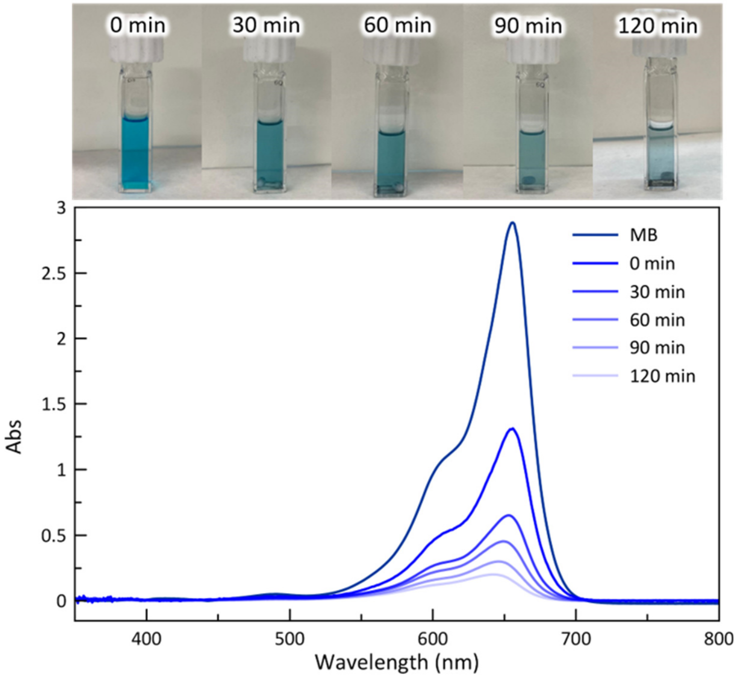

Figure S22. Methylene Blue photodegradation process. Cuvettes images during the reaction time in light condition and absorbance spectra of MB during the reaction time in light condition, the photocatalyst was removed by centrifugation.

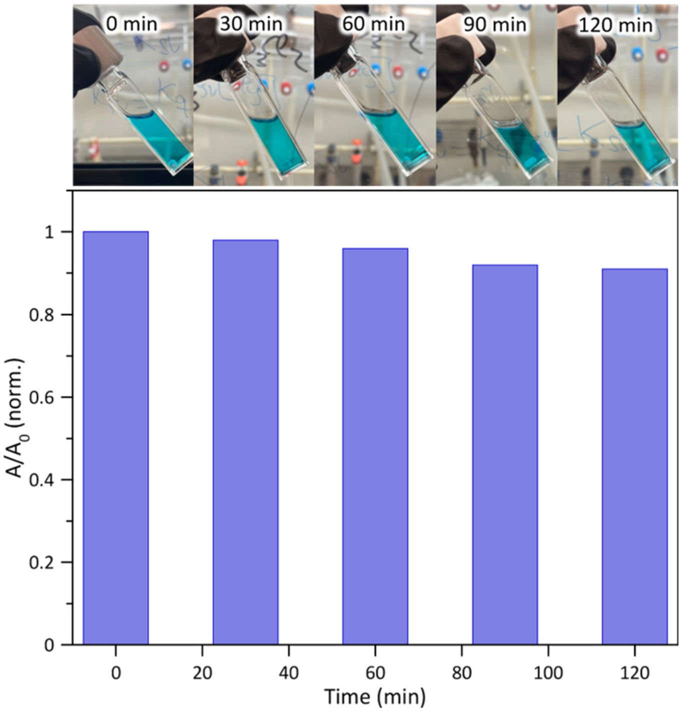

Figure S23. Methylene Blue test in dark condition. Cuvettes images during the reaction time in dark condition and normalized absorbance of MB during the reaction time in dark condition, the photocatalyst was removed by centrifugation.

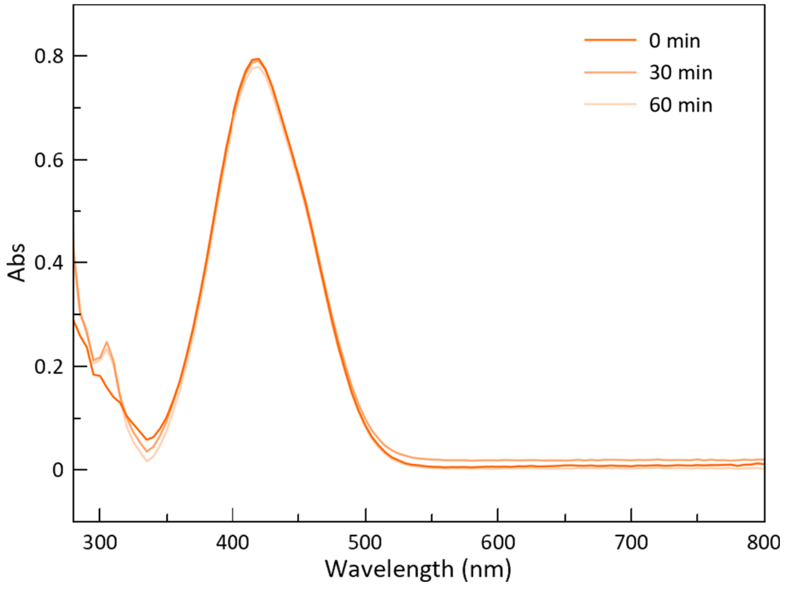

Figure S24. Methyl Orange photodegradation process. Absorbance spectra of MO during the reaction time in light condition, the photocatalyst was removed by centrifugation.

Table S1. Z potential of Bi/$Bi_{13}S_{18}Br_2$.

Name	Mean
Zeta Potential (mV)	-58,299
Conductivity (mS/cm)	0
Wall Zeta Potential (mV)	0
Quality Factor	1,037
Zeta Peak 1 Mean (mV)	-53,796

Open Circuit Voltage measures

Figure S25 gathers the OCP traces collected on control (a) and Bi HSs (b) samples. Upon illumination with a 415 nm light source, the open circuit voltage of control sample and heterostructures show a decrease in absolute value (i.e., the potential shifts towards more cathodic values). In both cases, this behavior is transient and reversible. Noteworthy, Bi HSs exhibit the noisier and complex trend (Figure S25b), with several spikes in the OCP value during the illumination. Nonetheless, a clear decrease/increase when the illumination is switched on/off is evident throughout the whole graph, thus confirming the interaction between Bi HSs and the light source.

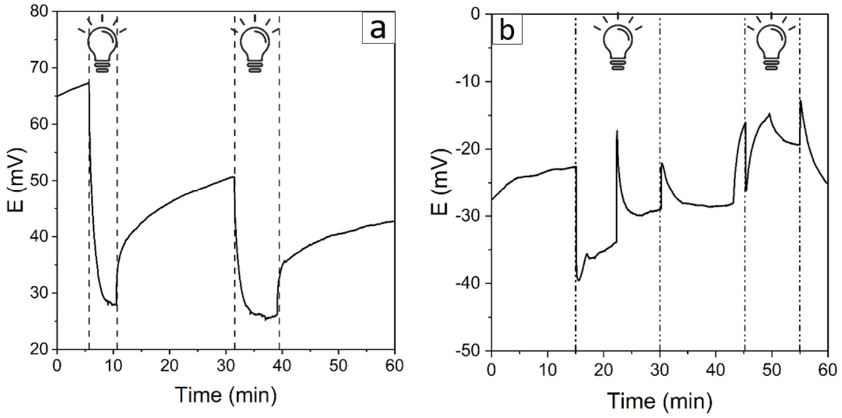

Figure S25. Open Circuit Voltage (OCP) traces. OCP traces recorded on (a) bare FTO, (b) Bi/ $Bi_{13}S_{18}Br_2$ HSs upon intermittent illumination with a 415 nm light source.

Cyclic Voltammetry (CV) of control samples

The CV collected on the bare support is gathered in Figure S26. As discussed in the main text, CVs do not show a significant variation upon illumination. This might be due to the limited contribution of the photogenerated currents with respect with the currents generated by the direct voltage application.

As for Bi HSs electrodes, CVs of bare FTO show additional voltammetric features when CO_2 is fed to the system, indicating a possible interaction of the material with CO_2. However, it must be noticed that the potentials screened in the bare FTO testing are way more cathodic than those investigated for Bi-based samples (Figure S25). This indicates a general inertness of FTO towards both HER and CO_2RR.

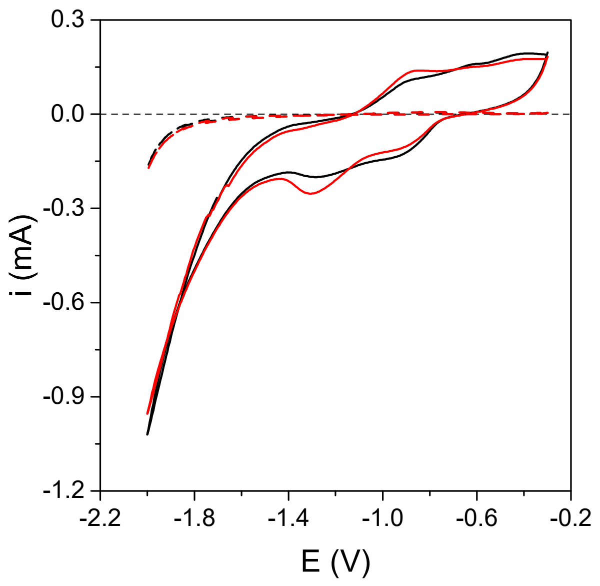

Figure S26. Cyclic Voltammetry (CV) traces. CV traces collected on bare FTO. (v = 100 mV s-1). Dotted curves have been collected under Ar bubbling (blank tests, only HER is possible), while full traces are related to CO2RR tests. Black: dark conditions. Red: illumination (415 nm LED). (b) CA scan at -1 V vs RE under intermittent illumination.

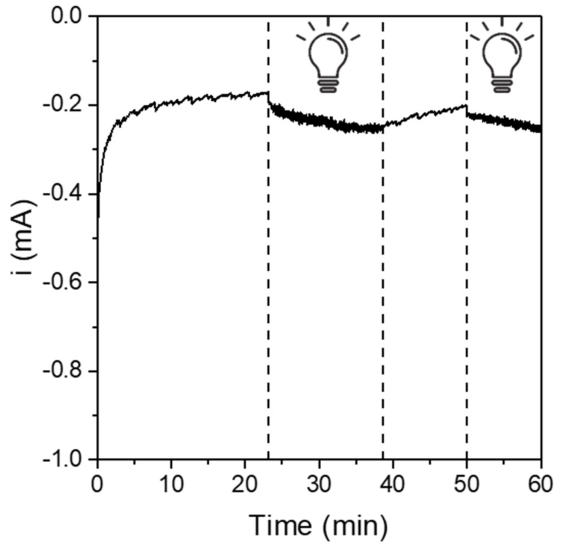

Figure S27: Chronoamperometry (CA). CA scans collected on bare FTO (@ -2 V vs RE) under CO_2RR conditions.

Gas and liquid-phase products detection

Despite the photoelectrochemical tests (especially those registered on Bi HSs electrodes) consistently indicate CO_2RR activity, the detection and quantification of products (also parasitic H_2, from HER) is hampered by the low currents delivered (i.e., low production rates). Indeed, the adaptation of our electrochemical CO_2RR setups, typically operated under flowing conditions, does not allow for the concentration of gas phase products in a static cell headspace, resulting in diluted samples, with concentrations lower than the instrument detection limit (Figure S28a). On the other hand, despite the expected concentration of liquid-phase CO_2RR products in the electrolyte, no products could be reliably quantified from HPLC chromatographs (Figure S28b).

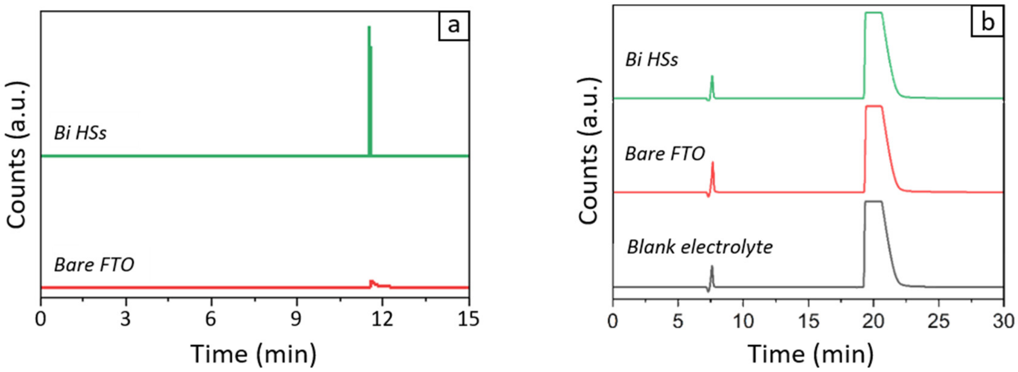

Figure S28. Gas chromatography and liquid chromatography. Sample chromatographic traces collected on the outlet gases and post-reaction electrolytes collected upon photoelectrochemical testing. (a) Typical FID (GC) and (b) RID (HPLC) traces.

[i] Quarta et al., “Colloidal Bismuth Chalcohalide Nanocrystals”, Angew. Chem. Int. Ed. 2022, 61, e202201747

[ii] Toso et al., “Structure Prediction of Ionic Epitaxial Interfaces with Ogre Demonstrated for Colloidal Heterostructures of Lead Halide Perovskites”, *ACS Nano* 2025, 19, 5, 5326–5341
